# LIKE EARLY STARVATION 1 and EARLY STARVATION 1 promote and stabilize amylopectin phase transition in starch biosynthesis

**DOI:** 10.1126/sciadv.adg7448

**Published:** 2023-05-26

**Authors:** Chun Liu, Barbara Pfister, Rayan Osman, Maximilian Ritter, Arvid Heutinck, Mayank Sharma, Simona Eicke, Michaela Fischer-Stettler, David Seung, Coralie Bompard, Melanie R. Abt, Samuel C. Zeeman

**Affiliations:** ^1^ Institute of Molecular Plant Biology, ETH Zurich, Universitätstrasse 2, 8092 Zurich, Switzerland.; ^2^ Université de Lille, CNRS, UMR 8576–UGSF–Unité de Glycobiologie Structurale et Fonctionnelle, Lille, France.; ^3^ Institute for Building Materials, ETH Zurich, Stefano-Franscini-Platz 3, 8093 Zurich, Switzerland.

## Abstract

Starch, the most abundant carbohydrate reserve in plants, primarily consists of the branched glucan amylopectin, which forms semi-crystalline granules. Phase transition from a soluble to an insoluble form depends on amylopectin architecture, requiring a compatible distribution of glucan chain lengths and a branch-point distribution. Here, we show that two starch-bound proteins, LIKE EARLY STARVATION 1 (LESV) and EARLY STARVATION 1 (ESV1), which have unusual carbohydrate-binding surfaces, promote the phase transition of amylopectin-like glucans, both in a heterologous yeast system expressing the starch biosynthetic machinery and in *Arabidopsis* plants. We propose a model wherein LESV serves as a nucleating role, with its carbohydrate-binding surfaces helping align glucan double helices to promote their phase transition into semi-crystalline lamellae, which are then stabilized by ESV1. Because both proteins are widely conserved, we suggest that protein-facilitated glucan crystallization may be a general and previously unrecognized feature of starch biosynthesis.

## Teaser

Two conserved non-catalytic proteins with unique carbohydrate-binding surfaces promote the phase transition of amylopectin.

## INTRODUCTION

Starch is the most abundant and widespread nonstructural carbohydrate in higher plants. In seeds, roots, and other perennating tissues, starch serves as a long-term carbohydrate reserve, fuelling germination or seasonal regrowth. This “storage starch” is a major constituent of our staple crops (e.g., cereals, potato, cassava, legumes, among others), accounting for half of human calorific intake, and is used extensively as an industrial commodity in the food, nonfood, and energy sectors (*1*). Starch is also synthesized in plant leaves during the day as a primary product of photosynthesis and is remobilized at night to sustain metabolism. This so-called “transitory starch” is very important for plant growth, as demonstrated by the reduced vigor of plants unable to make or degrade it effectively (*2*).

Starch is composed of two glucose polymers—amylopectin and amylose—and forms insoluble, semi-crystalline granules. The latter characteristic is conferred by the major component, amylopectin, a branched macromolecule consisting of linear chains of α-1,4–linked glucose units with α-1,6 branch points. The branched architecture is thought to give the molecule an overall tree-like appearance, featuring interconnected clusters of linear chain segments (*3*). Within this molecule, secondary and tertiary structures form, as pairs of adjacent chains entwine into double helices that align and pack into dense, crystalline lamellae. These crystalline lamellae alternate with amorphous lamellae harboring the branch points and the chains connecting the crystalline layers within the starch granule. Together, this regular arrangement of crystalline (~6 nm) and amorphous (~3 nm) layers is believed to underlie the 9- to 10-nm repeat structure that is commonly observed for plant starches (*3*, *4*).

Three major enzyme classes, working in an interdependent and concerted fashion, are involved in amylopectin biosynthesis: First, soluble starch synthases (SSs) elongate existing glucose chains by transferring the glucosyl moiety from the substrate adenosine diphosphate (ADP)–glucose (ADP-Glc), thereby forming an additional α-1,4 bond. Second, branching enzymes (BEs) create α-1,6 branches by transferring small glucan segments from an existing linear chain to the C6 position of another glucose unit elsewhere in the glucan molecule (*5*). Third, debranching enzymes of the isoamylase (ISA1) class hydrolyse some of the introduced branches (*6*). This presumably serves to remove surplus or misplaced branches that interfere with the formation of higher-order amylopectin structures and thereby facilitates amylopectin crystallization (*7*–*9*). This role of ISA1 is evident from the phenotypes of mutant plants lacking it; for example, in *Arabidopsis* (where ISA1 associates with a related, nonenzymatic ISA2 subunit to form a single heteromultimeric isoamylase), the loss of ISA1 activity causes a reduction in leaf starch together with the accumulation of substantial amounts of an aberrant soluble glucan called phytoglycogen (named according to its similarity to glycogen) in its place (*9*–*11*). Compared to amylopectin, phytoglycogen is enriched in short chains and has more branch points, which are located closer together, presumably because branches that remain would normally be removed by ISA1. Its suboptimal branching pattern is proposed to hinder the transition into a semi-crystalline state, and, as a consequence, phytoglycogen is soluble. Note although that besides phytoglycogen, *Arabidopsis isa1* mutants generate a variety of different glucans, including normal-looking starch granules, albeit with an altered amylopectin structure, in certain cell types (*9*, *12*).

Starch biosynthesis is highly conserved between plant species. Recently, the pathway from *Arabidopsis* was systematically reconstructed in the yeast *Saccharomyces cerevisiae* using a synthetic biology approach (*13*). After purging the yeast of its glycogen metabolic genes, combinations of the plant amylopectin biosynthetic enzymes were expressed, together with a bacterial enzyme providing the ADP-Glc substrate. Depending on the enzyme complement, the resulting strains produced different branched glucan structures. This allowed not only biosynthetic factors to be characterized in isolation and at greater depth but also actors and features promoting or limiting glucan phase transition to be identified. For example, strains expressing all SS and BE isoforms produced branched but soluble glucans, while strains expressing the same enzymes but in addition ISA1 and ISA2 also accumulated substantial amounts of starch-like insoluble glucans, reinforcing the key role of ISA1 in promoting the formation of semi-crystalline granules. While the insoluble glucans produced by this strain had many starch-like properties, some structural differences to plant amylopectin remained and not all glucans made the phase transition. One explanation for this is that the higher-order structuring of amylopectin may require more than just the known set of the starch biosynthetic enzymes.

In this study, we investigated whether two widely conserved, nonenzymatic plant proteins, LIKE EARLY STARVATION 1 (LESV) and EARLY STARVATION 1 (ESV1), could be involved in promoting the phase transition of amylopectin. Both proteins are found associated with starch granules from different species (*14*, *15*). In addition, *Arabidopsis esv1* mutants were identified in a genetic screen for plants that exhaust their starch reserves too fast at night and so display signs of early nighttime carbon starvation (*14*). Starch produced by *esv1* appears to have a normal glucan structure but seems more susceptible to degradation, such that it is turned over too fast not only at night but also even during the day, during net starch accumulation. In contrast, ESV1 overexpression causes excess starch to accumulate. The second protein, LESV, shares strong sequence homology to ESV1 in its C-terminal region, which is very rich in aromatic and acidic amino acid residues. The *lesv* mutant does not have altered nighttime starch degradation, but LESV overexpression causes the formation of many small starch granules and alters their turnover. Thus, the two proteins appear to be functionally related, albeit with distinct roles.

Here, we analyze the recently available AlphaFold predictions of the ESV1 and LESV protein structures and validate these predictions using small angle x-ray scattering (SAXS) and circular dichroism (CD) spectroscopy. We show that parts of the two proteins form a previously undescribed carbohydrate interaction surface capable of glucan binding. Using both a synthetic biology approach in yeast and in vivo experiments in *Arabidopsis*, we provide direct evidence that LESV promotes the phase transition of branched, amylopectin-like glucans. We propose a model whereby the carbohydrate interaction surface of LESV serves as a template upon which to align amylopectin double helices and that this nucleates the crystallization of lamellae, while ESV1 serves to stabilize this structure, once formed.

## RESULTS

### The tryptophan-rich regions of ESV1 and LESV are strongly conserved

**Fig. 1.**
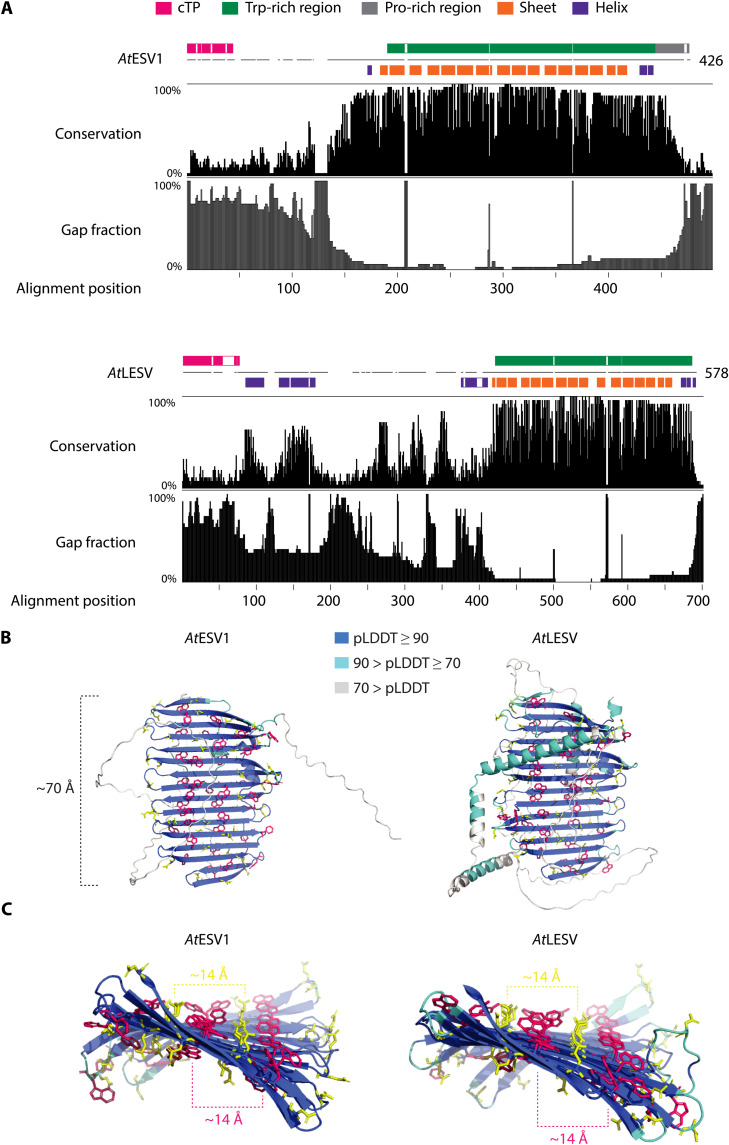
Structures and conservation of ESV1 and LESV proteins. (**A**) Conservation of homologous ESV1 and LESV protein sequences. Gap frequencies are based on ≥25 orthologous ESV1 and LESV sequences from (*14*) (for alignments, see data S1, A and B). Indicated in colored boxes above the *Arabidopsis* sequences are the chloroplast transit peptide (cTP), Trp-rich region, and Pro-rich region (ESV1 only) of the respective proteins. Colored boxes below represent the AlphaFold secondary structure predictions [see (B)]. Note that the alignment position does not correspond to the *Arabidopsis* protein residue numbering because of gaps. (**B**) AlphaFold predictions of ESV1 and LESV. Cartoon representations of the full-length proteins; the side chains of aromatic (Trp, Tyr, and Phe) and acidic residues (Asp and Glu) are shown in pink and yellow, respectively, and the protein backbone is colored according to confidence of the model (pLDDT value; see color key). (**C**) Isolated β sheet plane regions of ESV1 and LESV, reoriented to reveal the view along the length of the planes.

ESV1 and LESV were previously described to lack functional protein domains. However, the C-terminal ends of both proteins share a large region rich in aromatic amino acids, especially tryptophan (Trp), and the acidic amino acids aspartate and glutamate (*14*). To deepen our understanding of the proteins’ key characteristics, we retrieved orthologous sequences and assessed their conservation—an approach we deemed promising considering the recent demonstration of functional conservation for distantly related ESV1 homologs (*16*). Separate alignments of orthologous ESV1 and LESV proteins suggest that, in both cases, the entire Trp-rich regions identified earlier (*14*) are more stringently conserved than the remaining sequences, although additional regions of local conservation are revealed for LESV-like proteins (Fig. 1A). Within the strongly conserved C-terminal regions, the numerous Trp residues themselves are the most prominent sequence feature (fig. S1, A and B).

### The Trp-rich regions fold into unusual putative carbohydrate-binding surfaces

We next modeled the proteins’ tertiary structures, making use of the recently available AlphaFold platform (*17*, *18*). The Trp-rich regions of both ESV1 and LESV are predicted with high confidence to adopt peculiar conformations, folding into extended, twisted planar sheets, about 70 Å in length and consisting of 16 antiparallel β strands (Fig. 1B). The sequences N-terminal (including the putative transit peptides) and C-terminal to these β sheets are modeled with lower confidence scores; for ESV1, most of these parts are likely disordered and are poorly conserved (Fig. 1, A and B), including the proline-rich region previously noted (*14*). For LESV, several α helices are confidently [predicted local distance difference test (pLDDT) >70] predicted both N- and C-terminally of the β sheet. Because these are presumably flexibly connected to the β sheet via linker sequences, their position may vary depending on the protein’s state. While the N- and C-terminal parts of LESV are overall less conserved than the Trp-rich region, islands of conservation overlap well with some of the predicted α helices. Furthermore, three other conserved regions, one featuring a Trp pair, lie within the putatively unstructured protein parts (Fig. 1A and fig. S1B).

Given the high density of aromatic and acidic amino acids in the β sheets of ESV1 and LESV, we analyzed their spatial distribution. The aromatic residues aligned into two main strips spaced roughly 14 Å apart and running across both sides of the β sheet, perpendicular to the backbone β strands (Fig. 1, B and C). The acidic residues are chiefly located in the interstices between these aromatic strips, similarly organized in two strips also spaced 14 Å apart on both faces of the β sheet. Overlaying the ESV1 and LESV structures indicated that the distribution and orientation of aromatic and acidic residues are predicted to be extremely similar but not identical (fig. S2A). Some aromatic residues, specific to one or the other protein, are located either on the β strands at the level of the aromatic strips or on the loops connecting them.

To support the AlphaFold models experimentally, recombinant ESV1 and LESV proteins (minus transit peptides) were purified (fig. S2B) and analyzed for their secondary structure content by CD spectroscopy. Not only both proteins predominantly consist of β sheets, but LESV also had a certain fraction of α helices (fig. S2C), again consistent with the AlphaFold models. We next implemented SAXS to gather information on the three-dimensional (3D) shape of the proteins in solution. Both ESV1 and LESV showed smooth SAXS profiles, and analysis of the Guinier approximation plots confirmed that the proteins were nonaggregated (fig. S3, A and B, i to ii). Analyses of the protein particle sizes (table S1) and interatomic distance distributions showed that both ESV1 and LESV are composed of structured domains extended by elongated, more dynamic domains. We calculated 10 ab initio molecular envelopes for both proteins using DAMMIF (*19*), fitting the experimental data with χ^2^ of 1.7 (ESV1) and χ^2^ of 2.3 (LESV), and averaged and filtered them using DAMAVER/DAMFILT (*20*). The results (fig. S3, A and B, iii) suggest that both proteins contain oblate domains, whose dimensions and shapes are consistent with the shape of the conserved β sheets of the AlphaFold models.

It was previously proposed that the Trp-rich domain may be responsible for the association of ESV1 and LESV with starch granules (*14*). The Trp-rich regions alone were sufficient to mediate starch granule localization when transiently expressed in *Nicotiana benthamiana* (fig. S4). Together, these data strongly suggest that the Trp-rich regions of ESV1 and LESV constitute atypical carbohydrate-binding surfaces.

### ESV1 and LESV promote insoluble glucan accumulation in engineered yeast

**Fig. 2.**
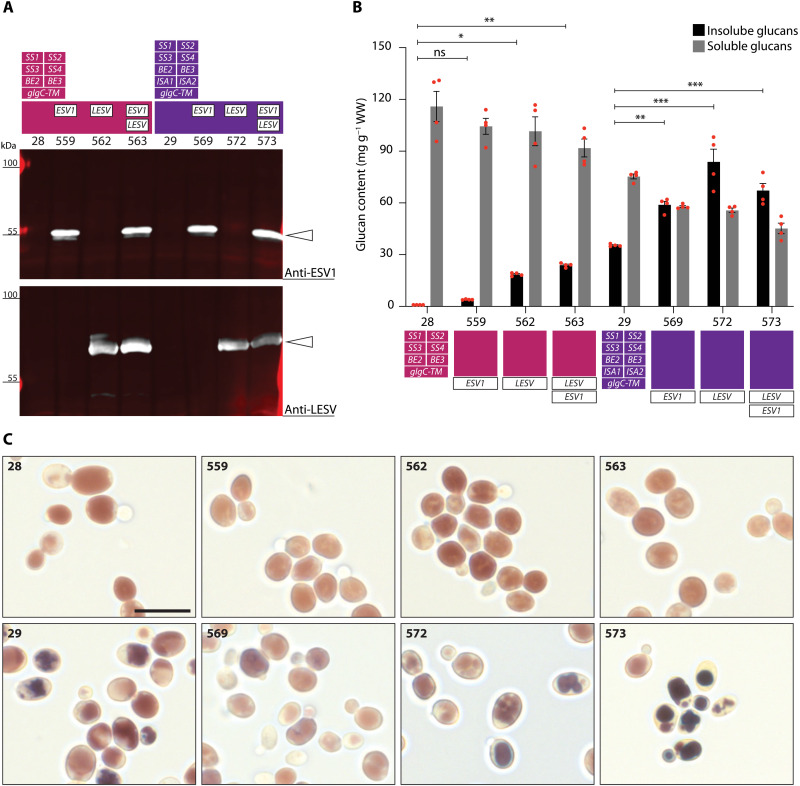
Expression of ESV1 and LESV in different yeast genetic backgrounds. (**A**) Immunoblots of total protein extracts from yeast strains expressing ESV1 (45 kDa), LESV (72 kDa), or both proteins in the 28 and 29 genetic backgrounds (expression sets indicated above the strain number) (*13*). ESV1 and LESV were detected using protein-specific antibodies in each case (white arrowheads). (**B**) Quantification of insoluble and soluble glucans of the yeast strains shown in (A), grown in liquid culture for 5.75 hours under inducing conditions. Values are means ± SE (*n* = 4); independent replicate cultures arose from different precultures. WW, wet weight. Statistical comparisons were performed using two-way analysis of variance (ANOVA) with Dunnett’s multiple comparisons test; see data S2 (A and B). Note that only comparisons for insoluble glucans to the respective parental strain are indicated in the graph. *P ≤ 0.05, **P ≤ 0.01, and ***P ≤ 0.001. ns, not significant. (**C**) Light micrographs (LMs) of iodine-stained yeast cells grown as in (B). For strain specifications, refer to (A) and (B). Scale bar, 10 μm.

To investigate the roles of ESV1 and LESV on starch biosynthesis in a simplified biological context, the proteins were expressed in *S. cerevisiae* cells previously engineered to synthesize starch-like glucans. Two yeast stains—designated lines 28 and 29—were used. Line 28 contains the *Arabidopsis* enzymes SS1 to SS4, BE2 and BE3, and accumulates large amounts of soluble glucan, while line 29 additionally contains the heteromultimeric isoamylase composed of ISA1 and ISA2, and a substantial fraction of its glucans are insoluble and semi-crystalline (*13*). Sequences encoding ESV1 and LESV (minus transit peptides), placed under the control of the *CWP2* promoter, were integrated by homologous recombination into the yeast’s nuclear genome individually or both together. Expression of the two proteins in the different strains was confirmed by immunoblotting (Fig. 2A).

We assessed the quantities of glucans accumulated by the strains, measuring the soluble and insoluble pools separately (the latter defined as sedimentable by centrifugation—see Materials and Methods). Expression of ESV1 in line 28 background (designated line 559) resulted in the accumulation of small amounts of insoluble glucans in addition to the soluble glucans typical of the parental line (Fig. 2B). This effect was even more pronounced when expressing LESV (line 562) or both proteins simultaneously (line 563). Similar effects were obtained when ESV1 and LESV were expressed in line 29, which already produces insoluble glucans. In this case, the additional expression of ESV1, LESV, or both (lines 569, 572, and 573, respectively) substantially increased the amounts of insoluble glucans to the extent that they exceeded the soluble glucans. Again, LESV expression or the simultaneous expression of both proteins had the greatest effect. In both backgrounds, the increase in insoluble glucans brought about by ESV1/LESV expression was associated with a commensurate decrease in soluble glucans, suggesting that the two proteins may influence the partitioning of glucans between the soluble and insoluble fractions rather than altering the extent of their accumulation per se.

### ESV1 and LESV influence insoluble glucan morphology in *S. cerevisiae*

Light microscopy (LM) was used to visualize the patterns of glucan accumulation in iodine-stained cells of the different yeast strains. As previously reported (*13*), cells of line 28 stained uniformly brown, as did ESV1-expressing line 559 (Fig. 2C). However, LESV-expressing lines (562 and 563) stained uneven, possibly reflecting the presence of both soluble and insoluble glucans. LM of line 29 revealed discrete, irregularly shaped particles—presumably the insoluble glucans. Intriguingly, in the ESV1-expressing line 569, the particles appeared larger and more regular in shape. This effect was even clearer in the LESV-expressing line 572 and most prominent in line 573, expressing both proteins. Together with the glucan fractionation and quantification, these data indicate that ESV1 and LESV promote the formation of insoluble glucans in our engineered yeast, acting irrespective of the presence of ISA.

**Fig. 3.**
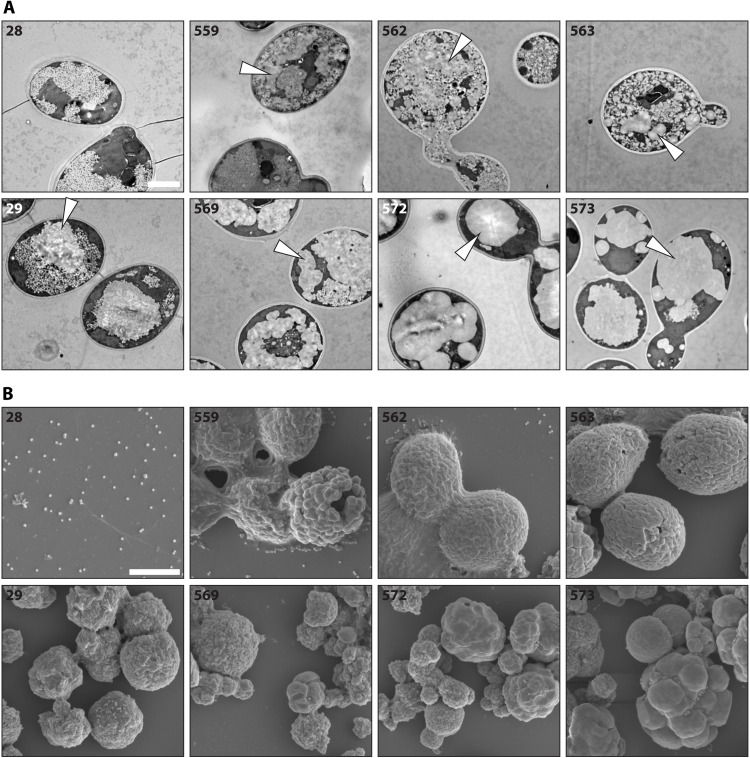
Appearance of soluble and insoluble glucans produced by yeast strains expressing ESV1 and LESV. (**A**) TEMs of the indicated yeast strains (for strain specifications, refer to Fig. 2). White arrowheads indicate presumably insoluble glucan structures. (**B**) SEMs of insoluble particles purified from the indicated yeast strains. Scale bars, 2 μm (A) and (B).

We performed transmission electron microscopy (TEM) of fixed and embedded cells (Fig. 3A) and scanning electron microscopy (SEM) of the purified insoluble glucans (Fig. 3B). As previously reported (*13*), TEMs of line 28 revealed large numbers of small, uniform glycogen-like particles in the cytoplasm. Consistent with this, no larger particles resembling insoluble glucans could be purified for SEM. Upon expression of ESV1, LESV, or both proteins (lines 559, 562, and 563, respectively), TEM revealed irregularly shaped deposits of particulate matter (Fig. 3A, arrowheads) alongside glycogen-like material similar to that in the parental line. Using SEM, the insoluble material from these three lines appeared as small, aggregated particles (Fig. 3B). The insoluble glucans in line 29 were visible by TEM as large, irregular particles alongside glycogen-like material in the cytoplasm and, by SEM, as granular structures with rough surfaces (Fig. 3, A and B) (*13*). The expression of ESV1, LESV, or both proteins (lines 569, 572, and 573, respectively) in line 29 substantially altered the morphology of the particles; many now appeared as aggregates of discrete, larger particles with smooth surfaces. This was evident from both TEMs (where less glycogen-like material was seen) and from SEMs of the extracted glucans and consistent with our LM observations (Fig. 2C). The influence on particle morphology, in terms of size and surface uniformity, was more pronounced upon expression of LESV compared with ESV1 but most obvious when both proteins were expressed together.

Comparing the glucans’ primary structure by their chain length distribution (CLD) profiles revealed that the primary glucan structures were similar to that of the parental lines (see fig. S5A and Supplementary Text). This is consistent with the absence of any known enzymatic function for ESV1 and LESV and suggests that their influence on glucan partitioning is not exerted by changing the glucan’s chain lengths or branch point frequency. Rather, it suggests that glucans transition more readily into an insoluble state. In yeast line 29, short malto-oligosaccharides (MOSs) accumulate besides soluble and insoluble, starch-like glucans owing to isoamylase action, which liberates chains from the branched glucans made by the SSs and BEs (*9*, *21*). Expression of ESV1 and/or LESV in line 29 substantially reduced the accumulation of MOS (again, this effect was strongest upon expression of LESV compared with ESV1; fig. S6), indicating that, in the presence of these proteins, a greater proportion of the glucan transitions into an insoluble form, with a lower degree of debranching by ISA.

### Aberrant starch formation occurs in the *lesv* mutant

**Fig. 4.**
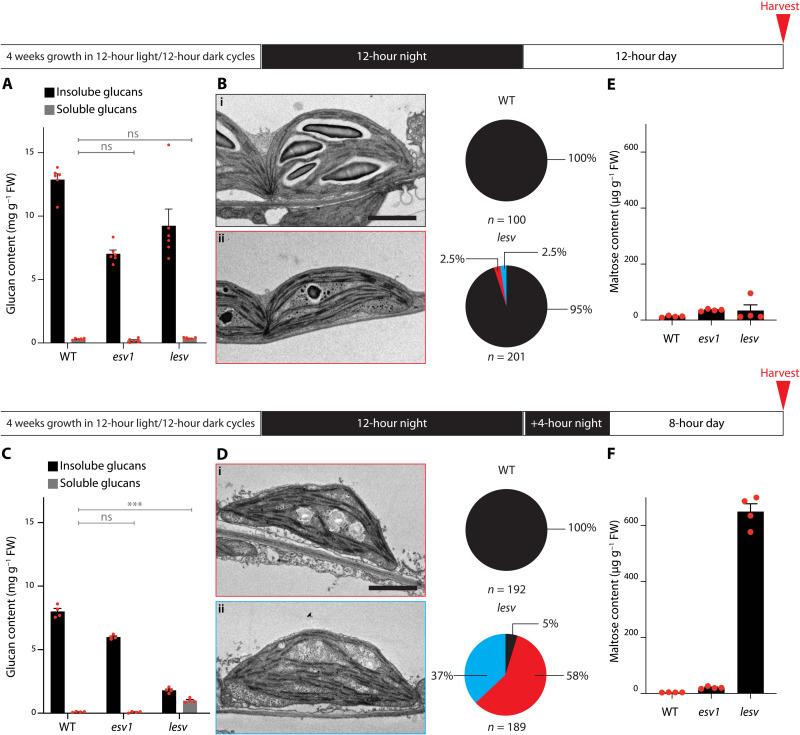
The *lesv* single mutant has a conditional mutant phenotype. (**A**) Glucan quantification of plants grown in 12-hour light/12-hour dark cycles, harvested as indicated. FW, fresh weight. Values are means ± SE (*n* = 6). Statistical comparisons were performed using two-way ANOVA with Dunnett’s multiple comparisons test; see data S3 (A and B). Only comparisons to the wild type for soluble glucans are shown. WT, wild type. (**B**) TEMs of selected *lesv* chloroplast sections, obtained from plants grown and sampled as in (A). Most *lesv* plastids appear wild-type like (i), but a few contain unusual glucans (ii). Scale bar, 2 μm. Pie charts show quantitative data of plastid section classifications; black, sections containing regular starch granules; red, sections containing starch and apparent phytoglycogen; blue, sections containing phytoglycogen-like inclusions only. (**C**) Glucan quantification of plants grown as in (A), subjected to a single prolonged night (16 hours), and harvested as indicated. Values are means ± SE (*n* = 4). Statistical comparisons were performed as in (A); see data S4 (A and B). Only comparisons to the wild type for soluble glucans are shown. ***P ≤ 0.001. (**D**) TEMs of *lesv* plants grown and harvested as in (C). Most chloroplast sections contain either a mixture of starch and phytoglycogen (i) or phytoglycogen-like inclusions only (ii). Scale bar, 2 μm. Pie charts indicate respective quantifications, as in (B). (**E** and **F**) Quantification of maltose in the plants in (A) (E) and (C) (F). Note that maltose is not included in the soluble glucans as measured in (A) and (C). Values are means ± SE (*n* = 4). Statistical comparisons were performed using one-way ANOVA with Dunnett’s multiple comparisons test; see data S5 (A and B).

Next, we investigated the roles of ESV1 and LESV *in planta*. *esv1* mutants were initially identified via their tendency to degrade starch too quickly, resulting in early nighttime starvation. However, *lesv* mutants had normal starch levels and turnover, with a mutant phenotype only evident upon LESV overexpression (*14*), which partly contrasts with the observed impact of LESV on glucan partitioning in yeast. While glucan levels in the *lesv-1 Arabidopsis* T-DNA insertion line (hereafter referred to as *lesv*) appeared normal (Fig. 4A), and most *lesv* chloroplasts contained normal-looking starch granules, about 5% of the chloroplasts examined contained aberrant starch. Instead of normal lenticular granules, these plastids contained small granules with irregular surfaces surrounded by very small particles or just the small particles (Fig. 4B). These aberrant starch granules were not seen in the wild type nor in the *esv1-2* (hereafter referred to as *esv1*) T-DNA line.

We challenged the *lesv* mutant by subjecting it to a single prolonged night (16 hours in total) so as to completely destarch it, enforcing the re-creation of starch granules from scratch in the subsequent light period (*22*). After a subsequent 8-hour day, wild-type plants had produced near-normal levels of starch. However, *lesv* had a notable phenotype: It accumulated far less glucans in total, a substantial fraction of which was soluble (Fig. 4C). Visualization of these glucans by TEM showed that most *lesv* chloroplast sections contained a mix of aberrant granules and small, presumably soluble, particles (58%) or exclusively small particles (37%). Normal starch was observed in only 5% of chloroplast sections (Fig. 4D). The CLD profile of the starch was like wild-type starch, but that of the soluble glucans was enriched in very short chains (length < 6 glucose units; see fig. S5B and Supplementary Text). These short chains are indicative of amylolytic attack (*9*), and, indeed, the daytime level of maltose (the product of β-amylases) was greatly increased in *lesv* following the destarching treatment but not under normal conditions (Fig. 4, E and F). These data suggest that LESV strongly facilitates the process of de novo formation of insoluble starch granules. However, in regular day-night cycles, this deficiency is compensated for by other factors.

### Overexpression of ESV1 or LESV promotes starch accumulation in *Arabidopsis* isoamylase mutants

**Fig. 5.**
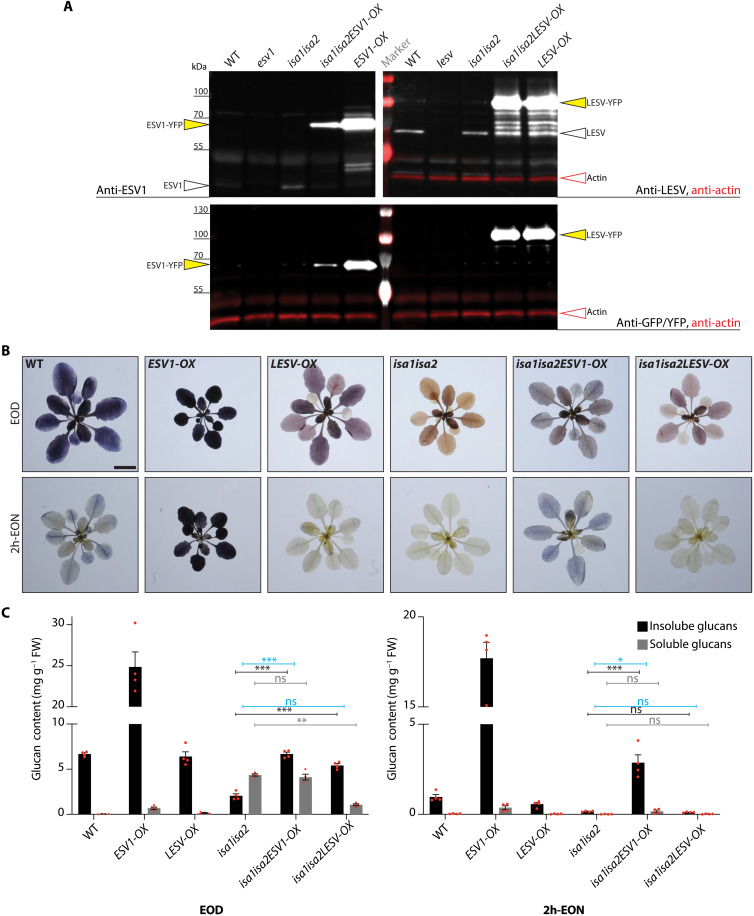
Glucan accumulation and turnover in plants overexpressing ESV1 and LESV in the *isa1isa2* background. (**A**) Endogenous ESV1 and LESV and overexpressed ESV1-YFP and LESV-YFP, as assessed by immunoblotting of total leaf protein extracts. Actin (in red) served as a loading control (omitted in the anti-ESV1 immunoblot for clarity). (**B**) *Arabidopsis* rosettes harvested at the EOD and 2 h-EON stained with Lugol’s solution. Scale bar, 1 cm. (**C**) Glucan quantification of plants harvested at the EOD and 2h-EON. Values are means ± SE (*n* = 4 biological replicates). Statistical comparisons were performed using two-way ANOVAs with Dunnett’s multiple comparisons test. Comparisons of total summed glucans are indicated in blue, those of soluble glucans (phytoglycogen) in gray, and those of insoluble glucans (starch) in black. *P ≤ 0.05, **P ≤ 0.01, and ***P ≤ 0.001. For clarity, only selected comparisons are shown. See data S6 (A and B).

To further reveal the impact of ESV1 and LESV on glucan crystallinity, we modulated their expression in the *isa1isa2* double mutant background. This isoamylase-deficient mutant predominantly makes soluble phytoglycogen in mesophyll cell chloroplasts but still produces some starch in epidermal and bundle sheath cells (*9*). We reasoned that this mutant background may represent an intermediate setting between our previously used yeast system and wild-type plants. Previously characterized *Arabidopsis* lines overexpressing yellow fluorescent protein (YFP)–tagged versions of ESV1 or LESV (*ESV1-OX*#3-2 and *LESV-OX*#4-6, hereafter referred to as *ESV1-OX* and *LESV-OX*, respectively) (*14*) were crossed to *isa1isa2*, and plants homozygous for the mutant and overexpression loci were identified. Immunoblotting confirmed the overexpression of ESV1-YFP and LESV-YFP in *isa1isa2ESV1-OX* and *isa1isa2LESV-OX*, respectively (Fig. 5A). For unknown reasons, ESV1-YFP was less abundant in *isa1isa2ESV1-OX* than in the *ESV1-OX* parental line. We reconfirmed granule association of these YFP-tagged protein versions by confocal microscopy (fig. S7) (*14*).

**Fig. 6.**
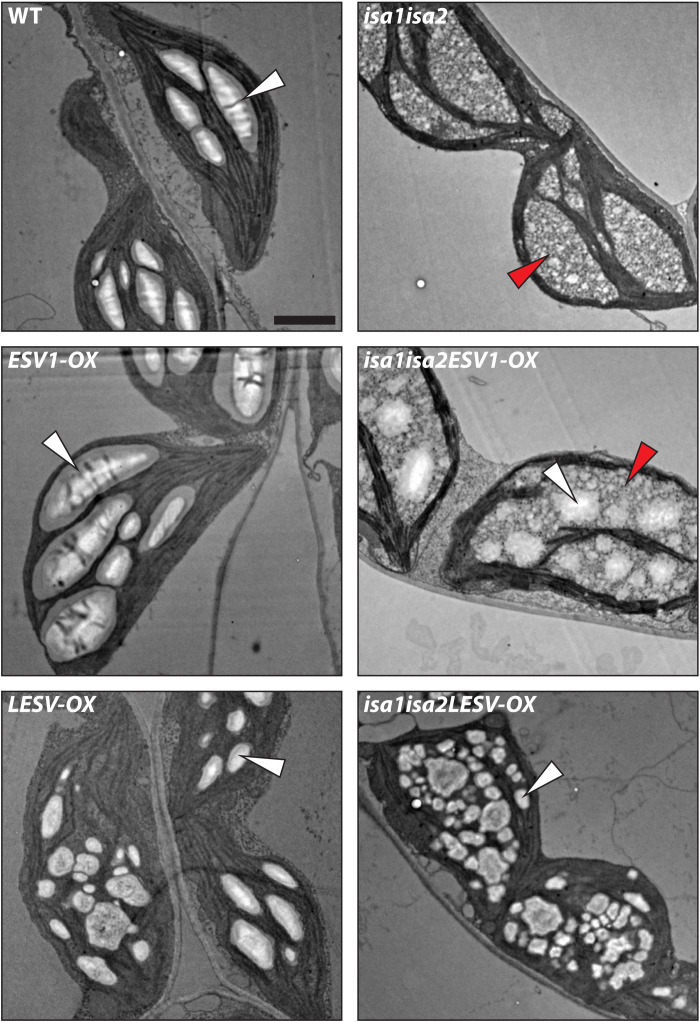
Appearance of glucans forming in mesophyll chloroplasts of plants overexpressing ESV1 or LESV in the *isa1isa2* background. Leaf tissue was harvested at the EOD, and the chloroplasts and starch granules therein visualized using TEM. White and red arrowheads indicate starch granules and phytoglycogen, respectively. Scale bar, 2 μm.

Next, these lines were characterized with respect to starch and phytoglycogen content at the end of the day (EOD). Consistent with previous reports, *ESV1-OX* had elevated starch contents and rosettes stained darker blue-black with I_2_/KI solution, indicative of its high amylose content (Fig. 5, B and C) (*14*). *LESV-OX* plants had similar starch contents as the wild type but stained less intensely, also consistent with its previously reported low amylose level. TEM confirmed that *ESV1-OX* had larger granules than the wild type in all cell types examined, while *LESV-OX* accumulated numerous small starch granules that were variable in appearance (Fig. 6) (*14*). The *isa1isa2* double mutant, with predominantly soluble phytoglycogen, stained orange-brown, and TEM analysis supported its previously reported cell-type–specific phenotype (Fig. 6 and fig. S8A) (*9*).

Both *isa1isa2ESV1-OX* and *isa1isa2LESV-OX* lines stained a darker color than *isa1isa2* at EOD, but their phenotypes differed (Fig. 5B); *isa1isa2ESV1-OX* not only had similar amounts of phytoglycogen as *isa1isa2* but also had starch levels comparable to the wild type. However, while *isa1isa2LESV-OX* also had wild-type levels of starch, it had very little phytoglycogen. Thus, in both lines, the starch fraction predominated over the phytoglycogen (Fig. 5C). TEMs of leaf tissue yielded results consistent with these quantitative analyses; *isa1isa2ESV1-OX* had both phytoglycogen and large starch granules in its mesophyll cells (Fig. 6) but only starch granules in epidermal and bundle sheath cells (fig. S8A). In contrast, *isa1isa2LESV-OX* contained many small starch granules in the plastids of all cell types examined but very little phytoglycogen (Fig. 6 and fig. S8A). These phenotypes were consistent over the leaf sections, as determined by LM (fig. S8B).

Similar I_2_/KI staining and quantitative analyses were carried out 2 hours before the end of the night (2h-EON), when *esv1* mutant plants have already prematurely degraded their starch (*14*). As expected, wild-type plants still contained some starch and rosettes stained lightly (Fig. 5, B and C). *ESV1-OX* rosettes stained much stronger and had excess starch, consistent with a reduced rate of starch mobilization, while *LESV-OX* did not. In *isa1isa2*, both starch and phytoglycogen had been consumed by this time. *isa1isa2ESV1-OX* had degraded its phytoglycogen but retained much of its starch. In contrast, *isa1isa2LESV-OX* had degraded its starch and what little phytoglycogen it had. These data suggest that ESV1 seems to limit the degradation of starch, while having little impact on the amount pf phytoglycogen that accumulates. LESV appears to promote the formation starch instead of phytoglycogen, without limiting degradation.

Because both overexpression lines were made using YFP-tagged proteins, we used confocal microscopy to examine the proteins’ distribution. *ESV1-OX* and *LESV-OX* lines yielded YFP fluorescent patterns consistent with starch granule localization (fig. S7) (*14*). In both *isa1isa2ESV1-OX* and *isa1isa2LESV-OX*, the fluorescence was primarily in the form of smaller, grouped punctae with a more diffuse background in areas between the chlorophyll autofluorescence, where glucans accumulate.

### Glucan turnover and crystallinity are affected by loss of ESV1 or LESV in *isa1isa2*

**Fig. 7.**
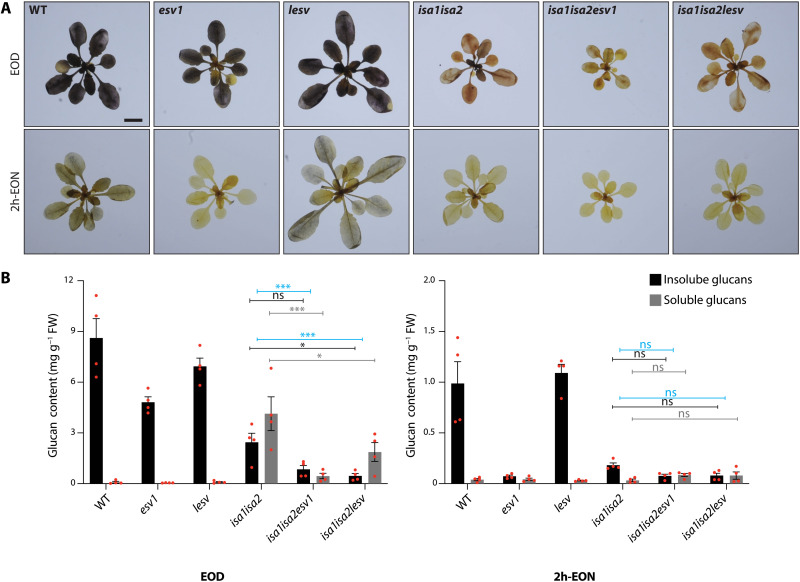
Glucan content of *isa1isa2*, *esv1*, *lesv*, and higher-order mutants thereof. (**A**) Entire rosettes were harvested at the EOD and 2h-EON and stained with Lugol’s solution. Scale bar, 1 cm. (**B**) Glucan quantification in plants, harvested at the EOD and 2h-EON. Values are means ± SE (*n* = 4 biological replicates). Statistical comparisons were performed using two-way ANOVA with Dunnett’s multiple comparisons test. Comparisons of total summed glucan contents are indicated in blue, soluble glucans (phytoglycogen) in gray, and insoluble glucans (starch) in black. For clarity, only selected comparisons are shown. *P ≤ 0.05 and ***P ≤ 0.001. See data S7 (A and B).

Next, we investigated the consequence of losing ESV1 or LESV function on the *isa1isa2* phenotype by generating the *isa1isa2esv1* and *isa1isa2lesv* triple mutants. At the EOD, the *esv1* and *lesv* mutants stained similarly to the wild type with I_2_/KI. However, *esv1* contained less starch than the other two lines (Fig. 7, A and B) (*14*). As before, *isa1isa2* accumulated predominantly phytoglycogen and less starch. Both the *isa1isa2esv1* and *isa1isa2lesv* triple mutants accumulated less glucan compared with their respective parental lines, with the reduction being particularly pronounced in *isa1isa2esv1.* However, the ratio of phytoglycogen:starch differed between the lines; in *isa1isa2lesv*, it was higher than in *isa1isa2*, while in *isa1isa2esv1*, it was lower (Fig. 7B). At 2h-EON, the wild type and *lesv* mutant still stained with I_2_/KI and contained a little starch, but *esv1* did not stain and contained almost none. The rosettes of *isa1isa2*, *isa1isa2esv1*, and *isa1isa2lesv* were also devoid of glucans at this time (Fig. 7, A and B), indicating that these lines had prematurely used their glucan reserves.

**Fig. 8.**
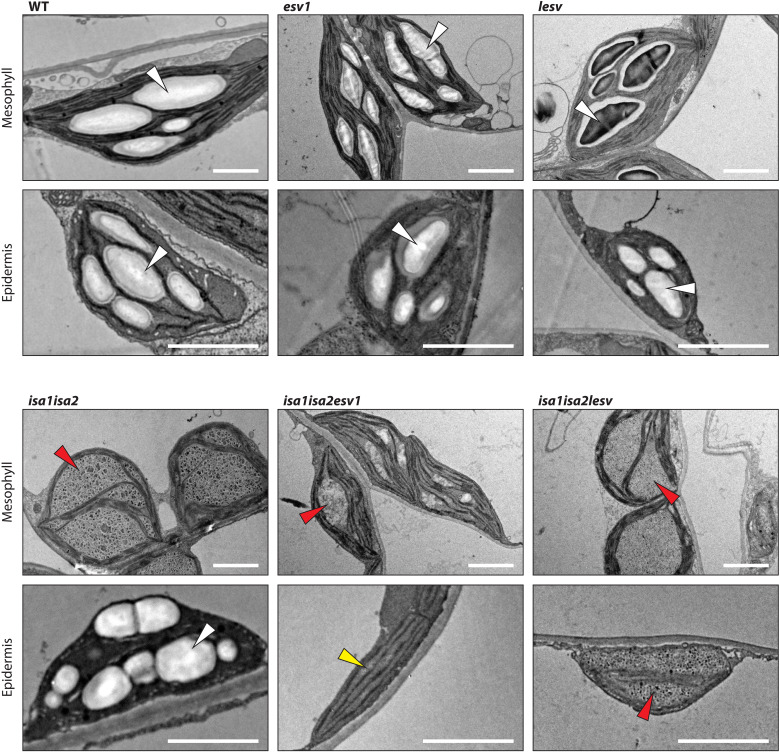
Glucans in mesophyll and epidermal cell plastids of *isa1isa2*, *esv1*, *lesv*, and higher-order mutants. Leaf tissue was harvested at the EOD, and plastids and starch granules observed using TEM. Arrowheads indicate starch granules (white), phytoglycogen (red), and plastid sections lacking glucans entirely (yellow). Scale bars, 2 μm.

To see how the cell-type–specific starch and phytoglycogen phenotype of *isa1isa2* was affected in the triple mutants, we investigated leaf mesophyll, epidermal, and bundle sheath cell plastids by TEM (Fig. 8 and fig. S9). As expected, mesophyll chloroplasts of *isa1isa2* contained phytoglycogen with few starch-like particles, while epidermal and bundle sheath cell plastids contained only starch granules. In *isa1isa2esv1*, the mesophyll cells contained less glucan, but a mix of starch and phytoglycogen was still evident; however, in epidermal cells, neither starch nor phytoglycogen was observed, consistent with the overall very low glucan content of this line (Fig. 7B). A contrasting result was obtained with *isa1isa2lesv*: The plastids in the mesophyll, epidermal, and bundle sheath cells all contained only phytoglycogen (Fig. 8 and fig. S9).

The accumulation of phytoglycogen, as opposed to starch, in *isa1isa2* is ultimately due to the aberrant structure of the glucan produced, yet overexpression or mutation of ESV1 or LESV shifted the starch:phytoglycogen ratio in a similar overall way as in engineered yeast cells (Figs. 2 to 6). We again tested whether this was due to a modified structure of the glucan by obtaining the CLDs for the glucans from each of the genetic backgrounds. As seen for the yeast, the absence or overabundance of ESV1 or LESV had only minor impacts on the glucan’s primary structure despite causing major changes in their turnover and/or partitioning between the soluble and insoluble phases (see fig. S5B and Supplementary Text).

**Fig. 9.**
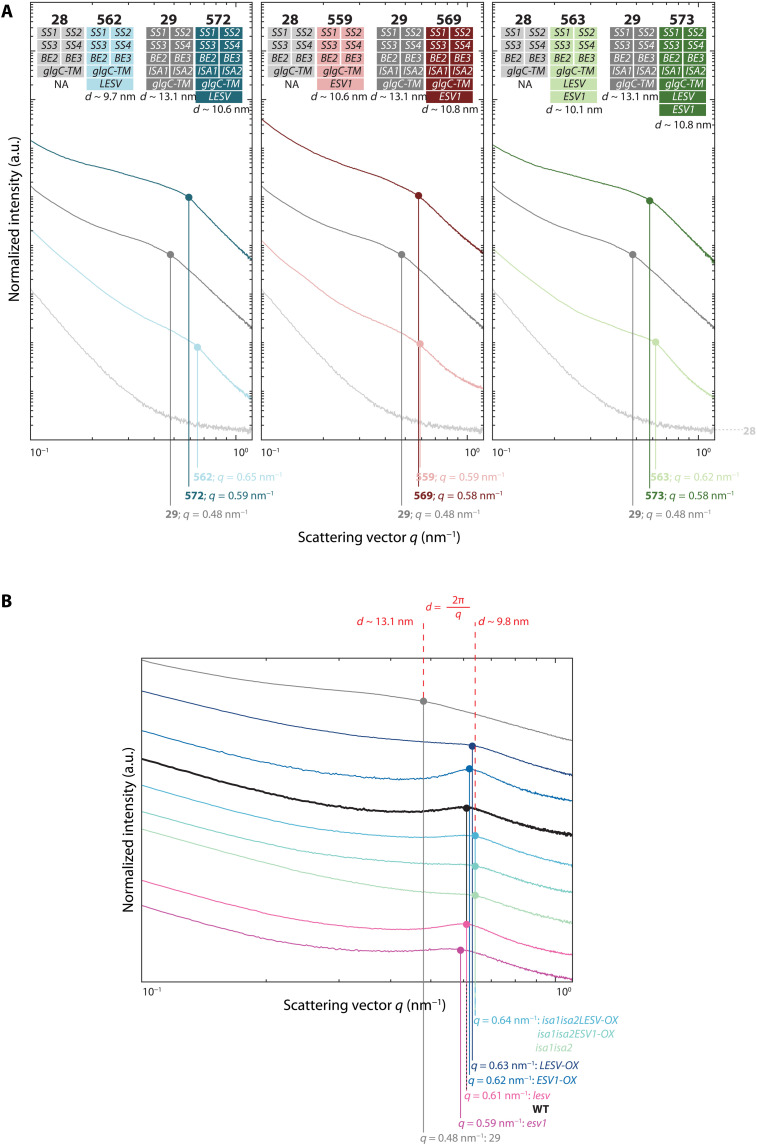
SAXS of purified yeast and plant glucans. (**A**) Stacked SAXS plots obtained from glucans purified from yeast strains expressing ESV1 and LESV. The local intensity maxima of individual samples were manually selected and are highlighted by colored dots. Vertical lines indicate the respective maxima’s *q* values. The respective calculated repeat distances (*d*) are indicated below the strains’ genetic descriptions. As expected, no maximum could be detected, and thus, no *d* was calculated, for yeast strain 28, due to the absence of insoluble glucans. NA, not applicable. (**B**) Stacked SAXS plots obtained from insoluble glucans purified from different plant genotypes. Displayed data are as in (A). In both (A) and (B), only one replicate measurement is shown; refer to table S2 for a summary of replicate analyses. a.u., arbitrary unit.

Last, we used small-angle x-ray scattering (SAXS) to investigate whether the presence of ESV1 or LESV influenced the lamellar structure of insoluble glucans. The starch-like glucans synthesized in yeast were shown previously to be enriched in long chains and to have a relatively weak lamellar repeat of over 13 nm compared to *Arabidopsis* amylopectin with its ca. 10-nm repeat (*13*). These observations were replicated here, with insoluble glucans from yeast line 29 giving a SAXS pattern consistent with a 13.1-nm repeat. The expression of ESV1, LESV, or both proteins in line 29 resulted in an altered SAXS pattern indicating a shorter repeat of 10.6 to 10.8 nm (Fig. 9A and table S2). Similarly, the insoluble glucans produced in line 28 expressing ESV1, LESV, or both proteins had short repeats (9.7 to 10.6 nm). Similar SAXS analysis applied to the starches extracted from plants showed that the loss or overexpression of either ESV1 or LESV did not change the lamellar repeat substantially, whether in the wild type or *isa1isa2* background: In all cases, the SAXS patterns were consistent with lamellar repeats in the region of 10 nm (Fig. 9B).

## DISCUSSION

The insight obtained from this work, using structural modeling and a set of complementary experimental approaches, provides a strong case for protein-mediated phase transition of amylopectin during starch biosynthesis. This is a paradigm-changing idea, since it has previously been assumed that phase transition is a biophysical process, occurring spontaneously when the amylopectin biosynthetic enzymes (SSs, branching, and debranching enzymes) generate a crystallization-competent structure. We suggest that, while these spontaneous crystallization can occur, it is promoted by the action of LESV. Furthermore, once a crystalline state is attained, we suggest that this state is stabilized by ESV1.

### ESV1 and LESV have unique starch binding domains

The ESV1 and LESV proteins were originally identified as starch-binding proteins (*14*). This property was proposed to be due to the unusual Trp-rich C-terminal domain that both proteins have, which we confirmed experimentally (fig. S4). Surface-exposed Trp residues, as well as the other aromatic amino acids (phenylalanine and tyrosine), are well known to form interaction sites for glucans, which bind via nonpolar CH-π staking interactions (*23*). The high-confidence structural models for the C-terminal domains of both ESV1 and LESV (Fig. 1), supported by our biophysical analysis of the recombinant proteins (fig. S3), are suggestive of a unique and remarkable protein-glucan interaction surface. The predictions for these domains are almost identical for both *Arabidopsis* proteins (fig. S2) and for several orthologs tested , consistent with the high degree of conservation between species (Fig. 1).

**Fig. 10.**
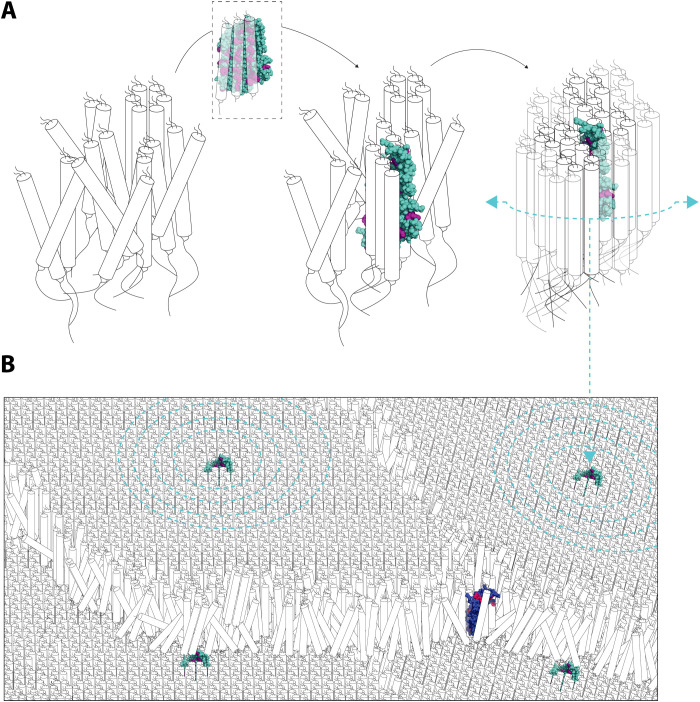
Model of the proposed ESV1 and LESV functions. (**A**) LESV interacts with glucans that have adopted a helical secondary structure via its Trp-rich domain and possibly aided by its N-terminal domain (not shown) and thereby facilitates their arrangement into compact, ordered tertiary structures. Once seeded, regular glucan arrangements can self-propagate and spread. (**B**) Regions of ordered glucan helices, seeded by LESV, form adjacent blocks of crystalline lamellae. ESV1 binds to and stabilizes exposed helices at the margins of these regions, restricting access to hydrolytic activities. In both (A) and (B), ESV1 and LESV proteins are represented as simplified versions of their predicted AlphaFold structures (only Trp-rich region is shown; aromatic residues are highlighted by color; pLDDT values are disregarded). See Fig. 1B as comparison.

The regular spacing of the side chains of ca. 30 of the 40 aromatic amino acids results in two strips adorning both sides of the extended antiparallel β sheet. Thus, extensive interactions with several glucan chains could occur. The β sheet domain has a length of approximately 7 nm, and the spacing of the aromatic amino acid strips is around 1.4 nm (Fig. 1, B and C). These dimensions are highly conspicuous, being similar to the predicted lengths and packing distances, respectively, of the double helices within the crystalline lamellae (*3*). We therefore propose that the strips of aromatic amino acids may coordinate the binding of neighboring double helices within an amylopectin molecule (Fig. 10). The frequent surface-exposed acidic residues (aspartates and glutamates) located between the aromatic residues could also participate in glucan binding by hydrogen bonding with the hydroxyl groups of the glucosyl residues (*24*).

Despite the similarity of their C termini, ESV1 and LESV differ substantially at their N termini, the structural predictions for which are less confident. No ordered structure is predicted by AlphaFold for the N terminus of ESV1, and it is notable that this part of the protein is poorly conserved between species. In contrast, parts of the N terminus of LESV are predicted to fold into α helices, consistent with our CD spectroscopy of the recombinant protein (Fig. 1 and fig. S2). Furthermore, there is overlap between this prediction and areas of high sequence conservation between species. That said, AlphaFold failed to predict ordered structure for a substantial part of the N terminus, and the predicted aligned errors for most of the N-terminal residues are high. Therefore, it seems likely that the exact organization of the helices, relative to each other and to the C-terminal domain, may differ to that shown in Fig. 1B. Two areas of conservation within the N terminus also feature prominent aromatic amino acids (fig. S1B), suggesting that it might also participate in glucan binding. Further studies will be needed to determine the structure of the N terminus of LESV, assess its binding capabilities, and see how these interrelate.

What is also clear, both from previous studies and from data presented here, is that the functions of the two proteins differ. The phenotypic effects of mutations abolishing expression of each gene, or overexpressing of each, are distinct (*14*). Furthermore, while aspects of the *esv1* mutant phenotype could be complemented by expression of the *Marchantia polymorpha* and *Oryza sativa* ESV1 orthologs, they could not by ectopic overexpression of LESV (*16*).

### A role for LESV promoting amylopectin phase transition

Collectively, our data suggest that LESV fulfills a previously unrecognized role in starch biosynthesis by promoting the phase transition of glucans. The evidence comes from several directions. First, in yeast cells engineered to make starch-like glucans, LESV is able to increase the fraction that transitions into an insoluble state, over that which crystalizes spontaneously. The resultant granules were larger and more uniform in appearance. The ability of LESV to promote phase transition was even seen in yeast cells where the complement of starch biosynthetic enzymes was incomplete, i.e., in line 28 where isoamylase is missing and where the resultant polymer otherwise remained exclusively in the soluble phase (Figs. 2 and 3). Second, this influence of LESV was also seen in isoamylase-deficient plants, where its overexpression substantially decreased the amount of soluble phytoglycogen and increased the amount of starch (Figs. 5 and 6). Consistent with this, the residual starch made in the *isa1isa2* double mutant was largely absent in the *isa1isa2lesv* triple mutant, suggesting that the endogenous LESV already promotes starch formation despite the suboptimal amylopectin branching pattern (Fig. 7). Third, starch formed after an extended night is highly abnormal in the *lesv* single mutant, with a substantial proportion remaining soluble as phytoglycogen despite the full complement of starch biosynthetic enzymes (Fig. 4). This result contrasts with the relatively mild phenotype of the *lesv* mutant that was reported previously (*14*) and was revealed only after destarching the plants.

Clearly, starch granules can form in the absence of LESV, but our results suggest that LESV promotes this by seeding glucan phase transition. We propose that the Trp-rich domain serves as a template to align double helices that form spontaneously between neighboring chains of amylopectin (Fig. 10A). We envisage that each strip of aromatic amino acids on each side of the β sheet domain could bind one double helix, and, once properly aligned on LESV, these double helices could then align others, propagating a wave of phase transition, resulting in a crystalline lamella. Given that LESV is found encapsulated inside starch granules—something also true for LESV orthologs from different species (*14*, *15*)—we assume that at least a fraction of it then remains associated with the crystalline lamellae. The seeding function may also require other parts of the LESV protein, including the conserved α-helical domains, or the regions with additional conserved aromatic residues but for which no structure is predicted.

This model could explain many of our observations. On one hand, when the glucan branching pattern is suboptimal (e.g., in the absence of the trimming isoamylase), the chance that double helices spontaneously self-align to seed a crystalline structure will be reduced, but the presence of a LESV template could allow it to occur. On the other hand, when the branching pattern is optimal, crystallization may occur spontaneously and self-propagate even in the absence of a LESV template. This is important, because it could explain the near-normal starch phenotype of the *lesv* mutant when growing under standard diel conditions (Fig. 4, A and B). During a normal night, most but not all starch is degraded (*2*). Any residual starch could itself serve as the template for the crystallization of freshly synthesized amylopectin the following day. Only when completely destarched, therefore, does the absence of both LESV and residual starch result in highly aberrant glucan formation (Fig. 4, C and D). It is also notable that, upon LESV overexpression, the number of starch granules is greatly increased. This could also be interpreted as accelerated crystallization, such that freshly synthesized soluble glucans assemble into more numerous distinct granules, rather than being added to the growing surface of existing granules.

Our SAXS analysis of the insoluble glucans adds a further angle of support for our proposed function for LESV (Fig. 9). The full suite of starch synthesizing enzymes in the yeast line 29 creates a crystallization-competent glucan, which spontaneously adopts a tertiary structure with a 13.1-nm repeat. However, the presence of LESV not only increased the amount that crystallizes but also shortened the lamellar repeat close to the 10-nm values seen for *Arabidopsis* starch. The 9- to 10-nm repeat in plant starch are formed by a crystalline (~6 nm) and amorphous (~3 nm) layer (*3*, *4*). The 7-nm length of the Trp-rich β sheet of LESV could thus guide the formation of double helices in this range, such that the next amorphous layer can be initiated on top of it. It would be interesting to see if engineering LESV to alter the length of its β sheet could influence the lamellar repeat length upon overexpression in yeast or in plants.

### A role for ESV1 in stabilizing semi-crystalline amylopectin

We propose a slightly different role for ESV1, which is to stabilize the amylopectin within the starch granule once it has formed, rather than to promote its crystallization in the first place. The evidence for this again comes from several directions. First, in yeast cells, ESV1 has a much smaller effect on promoting glucan phase transition compared with LESV (Fig. 2). Thus, while the Trp-rich domain of ESV1 seemingly can trigger phase transition, it is either less efficient than the equivalent domain of LESV or other parts of the LESV protein that ESV1 lacks also assist in the phase transition process (as discussed above). Second, when overexpressed in isoamylase-deficient plants, ESV1 increased the amount of starch but did not do so by decreasing the amount of soluble phytoglycogen (Fig. 5). Rather, the increase in starch is probably due to a block in its degradation at night; while the phytoglycogen is degraded, excess starch remains at dawn, leading to a higher overall glucan content. This excess starch accumulation is also seen when ESV1 is overexpressed in the wild type (*14*). Third, in the *esv1* mutant, starch is degraded too fast during the night and even concurrently with its accumulation during the day (*14*). However, no evidence for aberrantly formed granules or for soluble glucan accumulation comparable to the destarched *lesv* mutant was seen (Figs. 4, 7, and 8).

Given their semi-crystalline nature, starch granules are considered inherently resistant to enzymatic degradation. This view has been reinforced by the discovery that transient glucan phosphorylation is critical for normal degradation, with the reasoning that it disrupts the semi-crystalline structure (*1*, *25*–*29*). However, it is unlikely that the starch granule surface is uniform, and there may be areas where the structure is susceptible to degradation, regardless of phosphorylation. For example, at locations where one crystalline lamella ends and the next begins with a change in orientation, the double helices may be less stable. We envision that ESV1 could play a role at such sites, recognizing and stabilizing these weak points. Thus, in the absence of ESV1, these sites would be exposed, explaining the uncontrolled degradation in the *esv1* mutant. Conversely, ESV1 overexpression could render the granule more stable and even interfere with the process of phosphorylation, as proposed from in vitro work with recombinant ESV1 (*30*). Given this proposed role, we speculate that ESV1 may be primarily associated with the granule surface rather than becoming encapsulated like LESV. This is consistent with fewer ESV1 peptides being present inside starch granules compared to LESV (*14*). However, further experimental work will be required to test this hypothesis.

We suggest that soluble glucans, i.e., phytoglycogen, are less effectively protected by ESV1 than starch; all the phytoglycogen in *isa1isa2* is degraded at night, even when ESV1 is overexpressed (Fig. 5C). That said, phytoglycogen levels were very low in the *isa1isa2esv1* triple mutant (Fig. 7B), suggesting that in *isa1isa2*, ESV1 limits phytoglycogen turnover to some extent.

In conclusion, our work points toward a previously unrecognized biochemical process at play during starch biosynthesis. The amylopectin biosynthetic enzymes, working together on a common substrate, generate a glucan product with a propensity to crystallize. We propose that crystallization is facilitated by LESV and subsequently stabilized by ESV1. Clearly, this model needs further rigorous testing. The individual roles of each protein need to be defined, as do their potential combined effects when both are present and interacting with nascent glucans during starch biosynthesis or the mature granules themselves. The advantage of protein-mediated phase transition also needs to be established; it may help plants to make starch robustly in a wide variety of tissues and under different conditions. Variable factors such as temperature and plastid stroma solute content, i.e., during water stress, could differentially influence the starch biosynthetic enzymes, leading to alterations in amylopectin structure. Such an effect was reported for the starch in barley seeds developing at different temperatures (*31*). Furthermore, these variable factors may act as control parameters influencing the likelihood of spontaneous crystallization of the glucan. Thus, the presence of proteins that provide a template to nucleate crystallization and stabilize it could ensure efficient starch granule production and carbon storage even in suboptimal conditions. Last, the widespread conservation of both *ESV1* and *LESV* genes and the presence of the proteins in starches obtained from multiple sources (*14*) suggest that these results from *Arabidopsis* will be more broadly applicable in plants, including many of the world’s most important staple crops.

## MATERIALS AND METHODS

### Experimental design

The goal of this study was to better understand the molecular roles of the *Arabidopsis thaliana* ESV1 and LESV proteins in starch metabolism. This was approached from three angles: first, by studying the proteins’ sequences and 3D structures using bioinformatic methods and validating in vitro techniques using recombinantly expressed purified proteins; second, by studying the influence of ESV1 and LESV expression on glucan production and properties in an engineered yeast system; and, last, by investigating the effects of ESV1 and LESV overexpression or absence *in planta* using the *isa1isa2* double mutant as a sensitive genetic background.

### Bioinformatic analyses

For conservation analyses, orthologous streptophyte ESV1 and LESV sequences were extracted on the basis of the phylogenetic tree depicted previously (*14*) and aligned using Clustal Omega version 1.2.4. Alignments were annotated, and the conservation was depicted using CLC Genomics Workbench 12 (QIAGEN). Secondary structure predictions shown above the conservation plots are annotated on the basis of the respective *Arabidopsis* AlphaFold structures, irrespective of the respective regions’ pLDDT values.

WebLogos were constructed using WebLogo 3 (*32*) using compositional adjustment assuming a typical amino acid usage pattern. Predicted protein structures were obtained from AlphaFold [F4I9G2 (ESV1) and Q5EAH9 (LESV)] (*17*) and visualized using PyMOL (*33*).

### Cloning, expression, and purification of recombinant proteins

The coding sequence (CDS) of *LESV* (minus the 56 N-terminal residues predicted to encode the transit peptide), codon optimized for *Escherichia coli*, was cloned into the vector pET28a+ (Novagen), in-frame with an N-terminal 6XHis tag followed by a thrombin cleavage site, giving rise to the construct *pET28a-LESV*. Similarly, the codon-optimized CDS of *ESV1* was inserted into pET24a+ in-frame with a C-terminal 6XHis tag, giving rise to *pET24a-ESV1*. Because commonly used algorithms do not reliably predict a transit peptide for ESV1, we omitted 95 N-terminal residues, corresponding to a region that is poorly conserved in alignments of orthologous sequences.

*pET28a-LESV* was transformed into *E. coli* strain Rosetta (DE3) pLysS (Novagen). Precultures were grown overnight in LB medium supplemented with antibiotics at 37°C with agitation. For the main culture, ZY liquid broth supplemented with antibiotics was inoculated and incubated with agitation for 3 hours at 37°C, and bacterial cultures were then incubated overnight at 20°C. Cells were collected; resuspended in 50 mM tris buffer (pH 8), 300 mM NaCl, 10 mM imidazole, bovine pancreas deoxyribonuclease I (10 μg/ml), 20 mM MgSO4, and complete protease inhibitor cocktail EDTA-free tablets (one tablet per liter of culture; Roche) at 4°C; and then disrupted using an Avestin Emulsiflex disruptor. The bacterial extract containing soluble proteins was collected by centrifugation (10,000*g*) for 1 hour at 4°C. The extract was subjected to a first step of purification by immobilized metal affinity chromatography (IMAC) using His-Trap FP 5-ml column (Cytiva) equilibrated with 50 mM tris buffer (pH 8), 300 mM NaCl, and 10 mM imidazole. Recombinant protein was eluted using one step of 250 mM imidazole in equilibration buffer. This was followed by a second purification step by size exclusion chromatography using a HiLoad 16/60 Superdex 200 column (Cytiva) pre-equilibrated with 50 mM tris (pH 8), 150 mM NaCl, 10% (v/v) glycerol, and 2 mM dithiothreitol (DTT).

*pET24a-ESV1* was transformed into the bacterial strain BL21 codon+. Precultures were grown overnight in LB medium supplemented with antibiotics at 25°C with agitation. For the main culture, LB liquid broth supplemented with antibiotics was inoculated and incubated with agitation at 37°C to an optical density of 0.5 to 0.6 (*A*_600_), whereupon protein expression was induced by addition of isopropyl-β-d-thiogalactopyranoside (final concentration of 1 mM) for 3 hours at 37°C. Cultures were then incubated overnight at 20°C. Harvesting of bacteria and protein extract preparation were as described above. The extract was subjected to a first step of purification by IMAC using His-Trap FP 5-ml column (Cytiva) equilibrated in 50 mM tris (pH 7.5), 300 mM NaCl, and 40 mM imidazole. Bound protein was then eluted in one step in 50 mM tris (pH 7.5), 300 mM NaCl, and 250 mM imidazole. The protein solution was dialyzed against 50 mM tris (pH 7.5), 100 mM NaCl, 10% (v/v) glycerol, and 2 mM DTT.

### Synchrotron radiation CD

CD spectroscopy data were measured by synchrotron radiation CD at the DISCO beamline of the SOLEIL Synchrotron (Gif-sur-Yvette, France). Five microlitres of ESV1 at 6.3 mg/ml and 2 μl of LESV at 13.4 mg/ml were deposited between two CaF_2_ coverslips with a guaranteed pathlength of 20 and 10 μm, respectively (*34*). The beam size of 4 mm by 4 mm and the photon-flux per nanometer step of 2 × 10^10^ photons s^−1^ in the spectral band from 270 to 170 nm prevented radiation-induced damage (*35*). Spectra were collected consecutively over time and are the mean of 3 accumulations. The buffer baseline was recorded sequentially and subtracted from the spectra before taking into account the concentration in residues. Data were processed using CDToolX (*36*).

### SAXS of recombinant proteins

For SAXSs, recombinant protein samples were concentrated using Vivaspin centrifugal concentrators with a 10-kDa cutoff (Sartorius). Protein concentrations were determined using a NanoDrop Spectrophotometer (ND1000; Thermo Fisher Scientific). The maximum concentrations obtained for ESV1 and LESV for SAXS experiment are 4 and 21 mg ml^−1^ respectively.

Recombinant protein solutions were subject to centrifugation (10 min, 4°C, 10,000g) before x-ray analysis to eliminate potential aggregates. SAXS experiments were conducted on the SWING beamline at Synchrotron SOLEIL (λ = 1.033 Å). All solutions were mixed in a fixed-temperature (15°C) quartz capillary. The monodispersed samples of proteins were injected into a size exclusion column (SEC-3, 150 Å; Agilent) using an Agilent HPLC system and eluted directly into the SAXS flowthrough capillary cell at a flow rate of 0.2 ml min^−1^ (*37*). Protein samples (50 μl each) were then injected for SAXS measurements. During the first minutes of the elution, 150 frames were collected and averaged to account for buffer scattering, which was subtracted from selected frames corresponding to the main elution peak. Data reduction to absolute units, frame averaging, and subtraction were done using FOXTROT (*37*). All subsequent data processing, analysis, and modeling steps were carried out with programs of the ATSAS suite (*38*). The radius of gyration (*Rg*) was derived by the Guinier approximation using PRIMUS (*20*). The program GNOM (*39*) was used to compute the pair-distance distribution functions [*P*(*r*)] and feature the maximum dimension of the macromolecule (*D*max). 

### Cloning of yeast constructs and generation and growth of strains

Plasmids and primers used for cloning are provided in data S8. Constructs for heterologous expression in *S. cerevisiae* (yeast) were cloned using the protocols and toolkit for modular cloning in yeast (*40*). The CDSs of *ESV1* and *LESV*, less their putative chloroplast transit peptides, were cloned into pYTK001 (*40*) as detailed in data S8. The CDSs were introduced between the strong yeast *CWP2* promoter (pBP124) and *CYC1* terminator (pBP224) and assembled into yeast integration vectors designed for single transcription units (pBP386 and pBP387 in case of *ESV1* and *LESV*, respectively), resulting in the final expression vectors pBP389 and pBP392 for the expression of ESV1 and LESV, respectively, from the yeast locus XII-5 of the previously described yeast expression platform (*13*, *41*). For dual expression of ESV1 and LESV, both transcription units were assembled into the vector pBP249 (remaining positions were filled using primers mimicking the connector overhangs, added directly to the Bsm BI–mediated golden gate reaction; see data S8), which also targets the XII-5 locus, resulting in the final expression vector pBP393. Plasmids containing the CDS in pYTK001 were verified by sequencing. The correctness of the integration and final expression vectors was confirmed by diagnostic restriction digests using Pst I and Sac II.

*S. cerevisiae* strains derive from haploid CEN.PK113-11C. Strains expressing ESV1 and/or LESV were generated by transforming Not I–linearized expression vectors pBP389, pBP392, and pBP393 into yeast strains 28 or 29 (*13*), essentially using the transformation protocol described previously (*42*). Integration at the expected locus was confirmed by polymerase chain reaction (PCR) and amplicon sequencing as described previously (*13*). Genotypes of yeasts are given in table S3.

Media were prepared as described in (*43*), and yeasts were grown in shake flasks as described in (*13*). In brief, yeast cells from overnight cultures in YPD medium [1% (w/v) Bacto yeast extract (BD), 1% (w/v) Bacto peptone (BD), and supplemented with 2% (w/v) glucose] were inoculated in main cultures containing YPGal medium and harvested after 5.75 hours with shaking at 30°C. YPGal [which contains 2% (w/v) galactose] represents the inducing condition for all transgenes, except *ESV1* and *LESV*, which are driven by the *CWP2* promoter and thus constitutively expressed. Replicate main cultures arose from independent precultures.

### Plant materials and growth conditions

The mutants *esv1-2* and *lesv-1* (*14*), referred to as *esv1* and *lesv*, respectively, were crossed to *isa1-1isa2-1* (*9*), here referred to as *isa1isa2*, to generate the two triple mutants *isa1isa2esv1* and *isa1isa2lesv*. *isa1isa2* was also crossed to *ESV1-OX #3-2* and *LESV-OX #4-6* (*14*), referred to as *ESV1-OX* and *LESV-OX*, respectively, to generate *isa1isa2ESV1-OX* and *isa1isa2LESV-OX*. Plants homozygous for the respective mutations were identified by PCR-based genotyping (see table S4 for oligonucleotide primers). For the *LESV-OX #4-6* line, homozygous plants were identified by PCR-based genotyping, while for *ESV1-OX #3-2*, homozygous plants were identified by screening for BASTA resistance on plates. Wild-type (Col-0 ecotype) and mutant plants were grown as previously described (*21*). All mutant lines used in this study are in the Col-0 ecotype background.

### Protein extraction from yeast and plants and immunoblotting

Total protein extracts from yeast were prepared as previously described (*13*). Protein concentration was determined with a Bradford-based protein assay (Bio-Rad) using bovine serum albumin as standard. A total of 20 μg protein was loaded per lane for SDS–polyacrylamide gel electrophoresis (SDS-PAGE).

For total protein extracts from plants, leaves from 4-week-old rosettes were harvested and snap frozen in liquid nitrogen. Glass homogenizers were used to homogenize the plant material in extraction medium [1 ml/100 mg of fresh weight; 40 mM tris-HCl (pH 6.8), 5 mM MgCl_2_, 2% (w/v) SDS, and complete protease inhibitor (Roche)]. Insoluble debris was pelleted by centrifugation (5 min, 4°C, 20,000*g*) before loading equal sample volumes for SDS-PAGE.

For immunoblotting, proteins were transferred onto low-fluorescence polyvinylidene difluoride membranes following SDS-PAGE and probed with antibodies specifically recognizing ESV1, LESV, or green fluorescent protein (GFP). Antisera against *Arabidopsis* ESV1 and LESV (*14*) were used at concentrations of 1:200 for affinity purified anti-ESV1 and 1:3000 for anti-LESV crude serum. YFP-tagged proteins and plant actin (used as a loading control) were detected using commercial antibodies (anti-GFP: Torrey Pines Biolabs, TP401, at a concentration of 1:5000; anti-actin: Sigma-Aldrich, clone 10-B3, at a concentration of 1:10,000).

### Glucan extraction, quantification, and CLD analysis

For yeast, extraction protocols used were as described previously (*13*). Soluble and insoluble glucans were quantified using enzymatic assays after digestion to glucose, as described (*44*). For *Arabidopsis*, starch and phytoglycogen were extracted and quantified as described previously (*21*). CLDs of soluble and insoluble glucans extracted from yeast were obtained using protocols described previously (*13*). Similarly, CLDs of *Arabidopsis* starch and phytoglycogen were obtained using protocols described earlier (*21*).

### Iodine staining

Yeast cells from liquid cultures were stained with Lugol’s solution and imaged as described in (*13*). To stain *Arabidopsis*, rosettes were harvested at specified time points, decolorized in hot 80% (v/v) ethanol, rinsed in water, stained in Lugol’s solution for 3 min, and rinsed in water again before imaging.

### Scanning electron microscopy

For SEM imaging of insoluble glucans from yeast, glucans were purified using Percoll cushions as described earlier (*13*), coated with platinum, and imaged using an FEI Magellan 400.

### TEM and LM of embedded materials

For imaging yeast cells by TEM, protocols used were as described previously (*13*), with minor adjustments to the chemical fixation and gelling of cells before osmium staining. For the chemical fixation, cells grown in liquid culture in complex medium were prefixed with glutaraldehyde [50 mM sodium cacodylate (pH 6.8), 1 mM MgCl_2_, 1 mM CaCl_2_, and 2% (v/v) glutaraldehyde] for 1 hour at 20°C, pelleted by centrifugation (5 min, 20°C, 1500 *g*), and resuspended in the same fixative solution. After fixation using a BioWave (TedPella), the cells were kept at 4°C overnight. Cells were then pelleted and washed four times with water and once with 100 mM sodium cacodylate buffer (pH 6.8). Before post-staining with osmium, yeast cells were resuspended in water and mixed with one volume of 6% (w/v) low melting point agarose solution. After solidification, yeast cells were further processed, starting with osmium post-fixation as described earlier (*13*). Ultrathin (70 nm) sections were cut using a diamond knife, placed on formvar/carbon-coated copper grids, and stained with 2% (w/v) uranyl acetate and Reynold’s lead citrate. Images were acquired using a JEM-1400 Plus JEOL electron microscope.

To image *Arabidopsis* chloroplasts by TEM, young leaves of 4-week-old plants were harvested at EOD and cut into small pieces. These were fixed in 100 mM sodium cacodylate (pH 7.4), 2.5% (v/v) glutaraldehyde, and 2% (w/v) formaldehyde, followed by post-staining in 1% (w/v) osmium tetroxide, dehydration, and embedding into Spurr resin as described in (*22*) and sectioned and imaged as for yeast cells above.

Spurr blocks containing leaf tissue were also used for overview imaging by LM. Semi-thin (500 nm) sections were cut using a diamond knife, stained using toluidine blue O, and imaged using an AxioImager Z2 microscope (Zeiss).

### Cloning of ESV1 and LESV constructs for expression in tobacco

Sequences encoding full-length and truncated (Trp-rich regions only) ESV1 and LESV protein versions were amplified using constructs from (*14*) as templates. The resulting PCR products were first cloned into the pDONR221 vector and subsequently recombined into pB7WGY2 (for the truncated versions; in-frame with N-terminal sequence encoding the *Arabidopsis* Rubisco small subunit chloroplast transit peptide followed by sequence encoding YFP) or pB7YWG2 (for the full-length versions; in-frame with a C-terminal sequence encoding YFP) via gateway recombination cloning technology (Invitrogen), as described previously (*45*). The resulting constructs were transformed into *Agrobacterium tumefaciens*, infiltrated into tobacco (*N. benthamiana*), and the fluorescence was imaged as described previously (*14*).

### Confocal microscopy

Fluorescence from *ESV1-OX*, *LESV-OX*, *isa1isa2ESV1-OX*, and *isa1isa2LESV-OX* plants was imaged at end of the day as described previously (*46*) using a Zeiss LSM780 confocal imaging system, using either 514-nm (YFP) and 458-nm (CFP) argon or 633-nm (chlorophyll) helium-neon lasers. Image acquisition was done sequentially using filters ranging from 526 to 624 nm (YFP), 463 to 509 nm (CFP), and 647 to 721 nm (chlorophyll). At least two independent biological replicates were imaged per genotype, and imaging was repeated two or three times using different plant batches.

### SAXS of yeast and plant glucans

Yeast glucans for SAXS measurements were purified as described for SEM. For plant glucan purification, whole rosettes of individual plants were harvested and the material crushed with a pestle in extraction buffer [50 mM tris-HCl (pH 8), 2 mM EDTA, and 0.5% (v/v) Triton X-100] The slurry was sequentially filtered through wet nylon meshes with 150-, 30-, and 6-μm pore sizes. Glucans were then pelleted by centrifugation (10 min, 20°C, 6000 *g*). The resulting pellet was first washed with 0.5% (w/v) SDS until the supernatant was clear and then with water.

Approximately 20 μl of dense glucan suspension was injected into glass mark tubes (Hilgenberg) with an outer diameter of 0.7 mm and a wall thickness of 0.01 mm and sealed using a two-component epoxy resin. Glucans were allowed to settle before the capillaries were inserted into a laboratory SAXS system (Xenocs Xeuss 3.0). The scattering signal was recorded for the starch-enriched regions under vacuum using Cu Kα x-ray radiation (λ Cu Kα = 1.5419 Å) and a 2D detector (DECTRIS EIGER2 1M) positioned at a sample detector distance of 1300 mm. The recorded 2D scattering signal was azimuthally integrated to obtain the 1D scattering signal (scattering intensity as a function of the scattering vector). The background scattering from the capillary and water was scaled and then subtracted from the recorded signals to obtain the scattering signal solely from the starch. All data handling was performed using Xenocs XSACT software.

### Accession numbers

The *Arabidopsis* genome initiative gene codes for the *Arabidopsis* genes used in this study are the following: *ESV1*, *At1g42430*; *LESV*, *At3g55760*; *ISA1*, *At2g39930*; and *ISA2*, *At1g03310*.

## Notes

View/request a protocol for this paper from *Bio-protocol*.
