# Supplementary material for: LIKE EARLY STARVATION 1 and EARLY STARVATION 1 promote and stabilize amylopectin phase transition in starch biosynthesis
Source: Sci Adv. 2023 May 26;9(21):eadg7448. doi: 10.1126/sciadv.adg7448 (PMC10219597; doi:10.1126/sciadv.adg7448)
Supplement: Supplementary file 1 — Supplementary Text Figs. S1 to S9 Tables S1 to S4 Legends for data S1 to S8 [file sciadv.adg7448_sm.pdf]

Science Advances
AAAS

Supplementary Materials for

LIKE EARLY STARVATION 1 and EARLY STARVATION 1 promote and stabilize amylopectin phase transition in starch biosynthesis

Chun Liu *et al.*

Corresponding author: Samuel C. Zeeman, szeeman@ethz.ch

Sci. Adv. **9**, eadg7448 (2023)
DOI: 10.1126/sciadv.adg7448

The PDF file includes:

Supplementary Text
Figs. S1 to S9
Tables S1 to S4
Legends for data S1 to S8

Other Supplementary Material for this manuscript includes the following:

Data S1 to S8

Supplementary Text

Given the marked increase in insoluble glucans and the altered appearance of the particles upon expression of ESV1 and/or LESV, either in yeast cells or in the *isa1isa2* mutant background, we investigated whether there were changes to the glucans' primary structure, as revealed by their chain length distribution (CLD) profiles. Insoluble and soluble glucans from each line were debranched enzymatically and the resultant linear chains analyzed by HPAEC-PAD (Figure S5).

CLD analysis of glucans in the yeast lines:

The yeast line 28 synthesized soluble glucan that, compared to Arabidopsis amylopectin, was rich in short (DP4-7) and medium length (DP16-30) chains, and had fewer short (DP10-15) and long (DP>36) chains. Its structure was more similar – though not identical – to soluble phytoglycogen from the Arabidopsis *isa1isa2* double mutant. The CLDs of the insoluble glucans resulting from expression of ESV1, LESV, or both proteins in line 28, i.e. lines 559, 562 and 563, were remarkably similar to that of line 28, except for decreased numbers of very short chains (particularly DP5-7) and slightly increased numbers of longer chains. The soluble glucans, which still dominated in all four lines, were essentially identical.

The CLD of the glucans from line 29 differed from those of line 28, having fewer short chains (DP6-18) and more chains over DP25 (Figure S5A; (*13*)), presumably as a result of selective debranching by ISA. Line 29 has both soluble and insoluble glucans with the former having slightly fewer short chains (DP5-9) than the latter (Figure S5A; (*13*)). Upon ESV1 expression (line 569), the CLD of the insoluble glucan was unchanged. Upon LESV expression (line 572), or expression of both proteins together (line 573), there was a further slight decrease in the number of the short chains (particularly DP3-8) and slight increases in the number of longer chains in the insoluble glucans compared with line 29. There were also slight changes in the CLDs of the soluble glucans in these lines.

Overall, these data show that presence of ESV1 and/or LESV resulted in either no or minor changes in the structure of the glucans, with the CLDs most closely resembling that of the parental line in each case. We interpret these data to mean that the proteins do not directly alter the glucan structure. The small changes could be explained if ESV1 and/or LESV promote the transition of the glucan into the insoluble phase. On the one hand this might enrich for certain glucan structures, while on the other hand it might reduce the susceptibly of the now insoluble glucan to further enzymatic modification. It is also possible that the binding of ESV1 and/or LESV to a still soluble glucan could mask it and prevent other enzymes from using it as substrate.

CLD analysis of starch and soluble glucans accumulating in the Arabidopsis lesv mutant after de-starching with an extended night:

After an extended night, the *lesv* mutant accumulates a mixture of starch and soluble glucan the following day (Figure 4C). The starch CLD profile from this *lesv* mutant starch was essentially indistinguishable from that of the wild type (Supplemental Figure 5B). However, the soluble glucan had a different structure, with increased numbers of chains of DP3-8 and a decrease in most chains of DP11 and longer. These changes are reminiscent of the soluble phytoglycogen of *isa1isa2* (*9*). The marked increase in chains, especially those shorter that DP6, are a strong indicator of amylolytic degradation, since the shortest chain transferred by branching enzymes during amylopectin biosynthesis is DP6 (*5*). This is supported by the very high levels of the β-amylase degradation product, maltose, in the *lesv* mutant under these conditions (Figure 4E).

CLD analysis of glucans in the Arabidopsis lines modulated for ESV1, LESV and the ISA1/ISA2 debranching enzyme:

Starch extracted at the end of a normal day from the wild type, and from the *esv1* and *lesv* mutants had CLD profiles that were indistinguishable from each other (Figure S5C), consistent with earlier findings (*14*). In *isa1isa2* starch, however, there was a slight increase in chains of DP5-8 and a decrease in chains of DP12-16. These changes were more pronounced in the soluble phytoglycogen of *isa1isa2*, also as previously described (*9*).
The overexpression of ESV1 and LESV, in *isa1isa2ESV1-OX* and *isa1isa2LESV-OX* respectively, hardly altered the CLD profile of starch from *isa1isa2* - only chains of DP5-8 were slightly decreased in abundance. The phytoglycogen CLDs from *isa1isa2ESV1-OX* and *isa1isa2LESV-OX* were identical to that of *isa1isa2* (Figure S5C). The loss of ESV1 in the *isa1isa2esv1* triple mutant caused a minor increase in short chains (DP5-9) in the starch CLD, relative to *isa1isa2*. The loss of LESV in the *isa1isa2lesv* triple further enriched these short chains in the starch and resulted in fewer chains of DP12-16. This CLD was conspicuously similar to that of the phytoglycogen, and, since *isa1isa2lesv* accumulated very little starch (Figure 6A and B), may represent some phytoglycogen contamination of the insoluble material. The CLDs of the phytoglycogen, were more similar between the three genotypes (Figure S5C).
As with the yeast glucans, the CLDs of starch and phytoglycogen in these plants is unaltered or altered only to a minor degree depending on whether ESV1 and LESV are present.

A

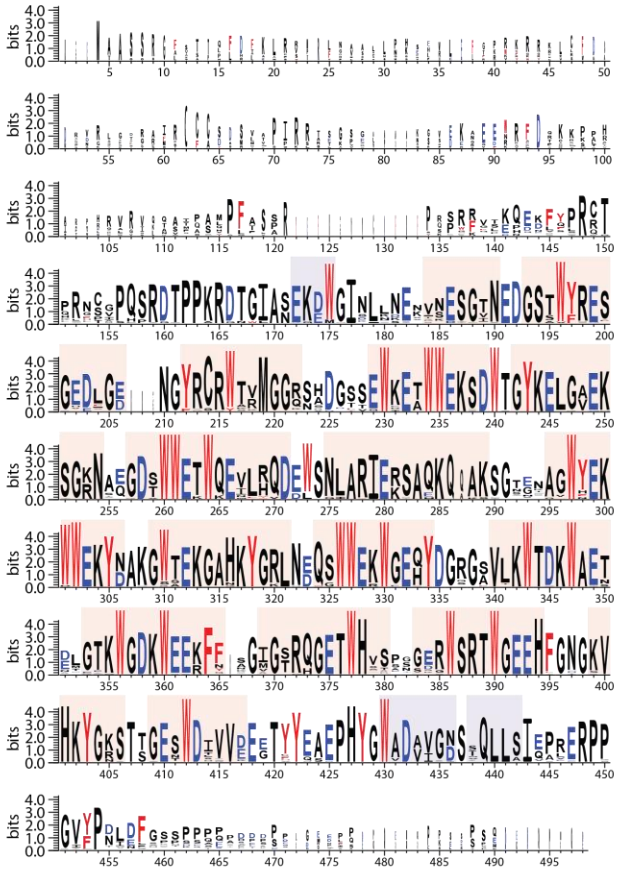

B

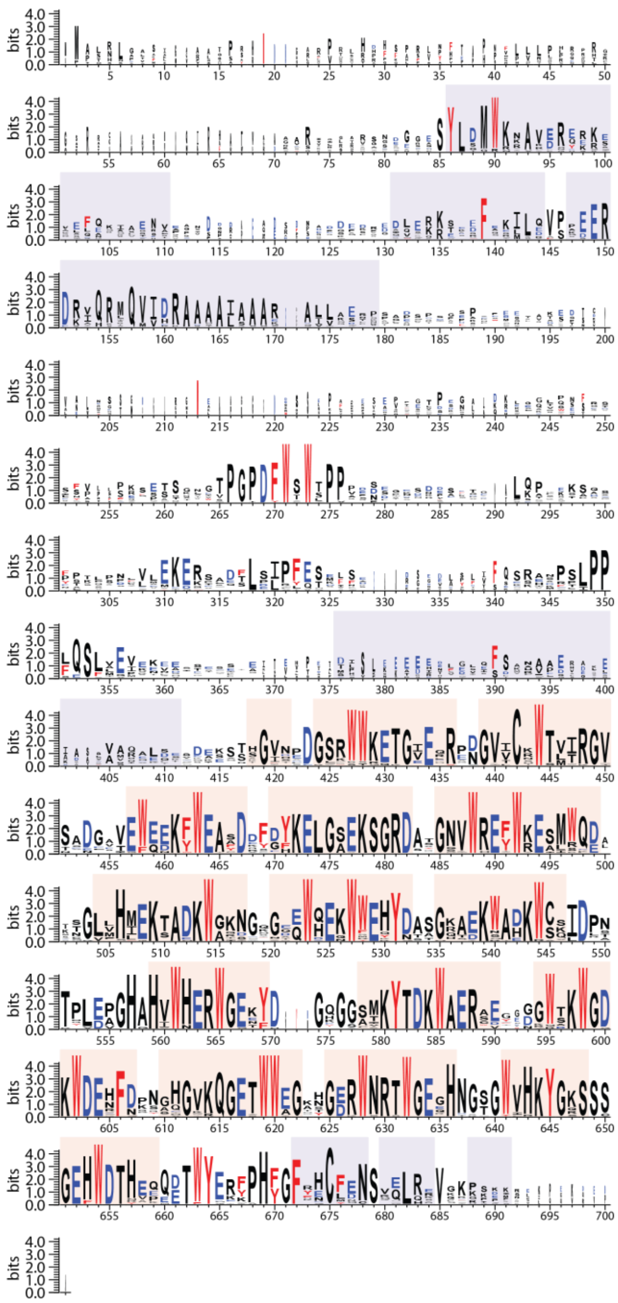

Fig. S1. Conservation hotspots in ESV1-like and LESV-like sequences. Displayed are WebLogos of the multiple sequence alignments of orthologous ESV1 (**A**) and LESV (**B**) sequences (used to create main Figure 1A). Aromatic amino acids are colored red, acidic ones blue. Underlaid in transparent boxes are the AlphaFold secondary structure predictions for the respective Arabidopsis protein, mapped to the alignment (coloring as in Figure 1A).

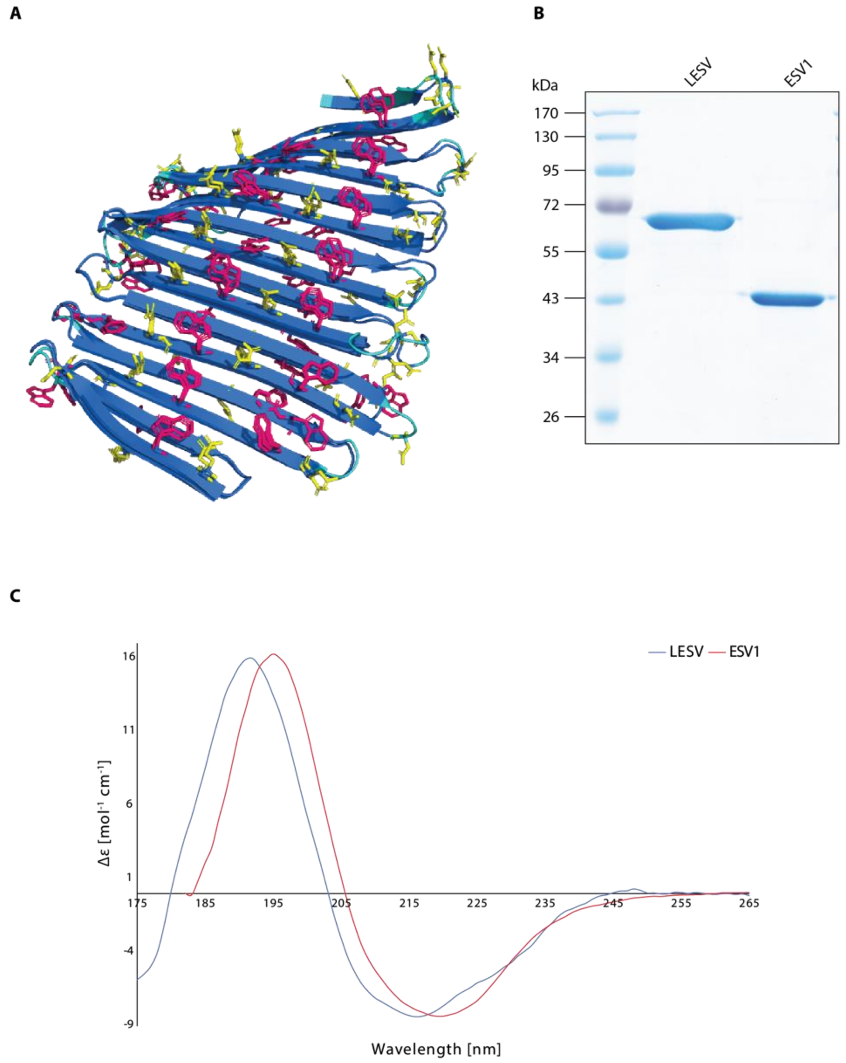

Fig. S2. The ESV1 and LESV beta-sheet planes. (**A**) Shown is a closeup of the overlaid ESV1 and LESV beta-sheet planes (as displayed in main Figure 1C). (**B**) Coomassie blue stained 10% SDS-PAGE. For both LESV and ESV1, 2 µg of the purified protein samples used for SAXS and CD experiments were loaded. (**C**) Far UV SRCD spectra of recombinant ESV1 and LESV proteins. Spectra represent the mean of three independent acquisitions. Only data with a HT (High Tension) of the photomultiplier tube > 400 V are represented.

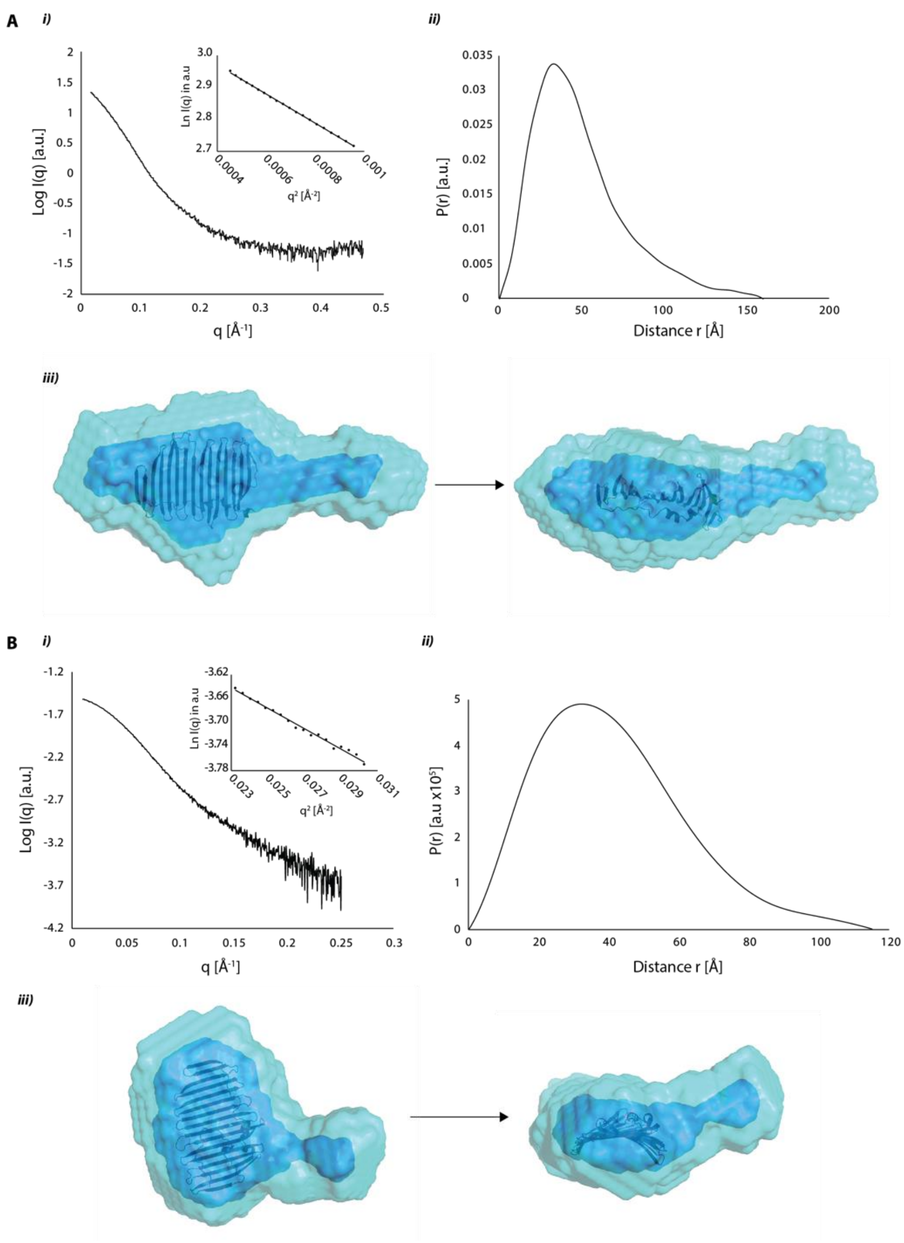

Fig. S3. SAXS data and *ab initio* models for recombinant LESV (A) and ESV1 (B) proteins. *i*) Experimental data plotted as a function of the scattering vector q (the Guinier plot is represented as an inset), *ii*) Distance distribution function and *iii*) Average *ab initio* envelope predicted by DAMMIF/DAMAVER (from 10 *ab initio* protein models computed by DAMMIF, in light cyan) superposed with the most typical shape filtered by DAMFILT (navy blue). The proteins' structural parts modeled with a confidence score (pLDDT) ≥ 90 (cartoon) are manually fitted into the molecular envelope. a.u., arbitrary unit.

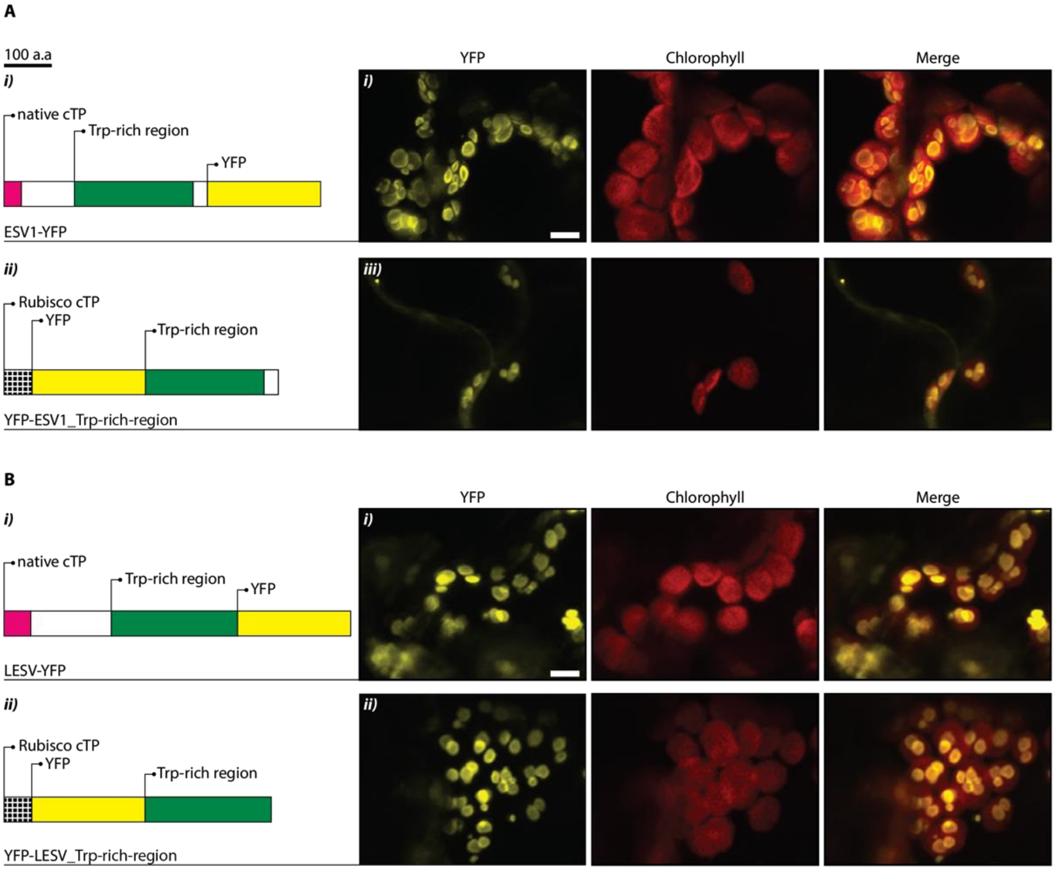

Fig. S4. Sub-plastidial localization of full-length and truncated ESV1- and LESV variants. Full-length and N-terminally truncated, YFP-tagged variants of ESV1 (**A**) and LESV (**B**) were transiently expressed in *Nicotiana benthamiana* leaves and their localizations determined by confocal microscopy. The respective protein variants' domain structures (*i-ii* for both ESV1 and LESV) are schematically depicted on the left of the confocal images (scale indicated in A is valid for B, too; a.a., amino acid residues). The Arabidopsis Rubisco small subunit cTP was used for the N-terminal YFP fusions. Presence of the Trp-rich regions alone is sufficient to mediate starch granule binding in either case. Scale bar, 5 µm.

A

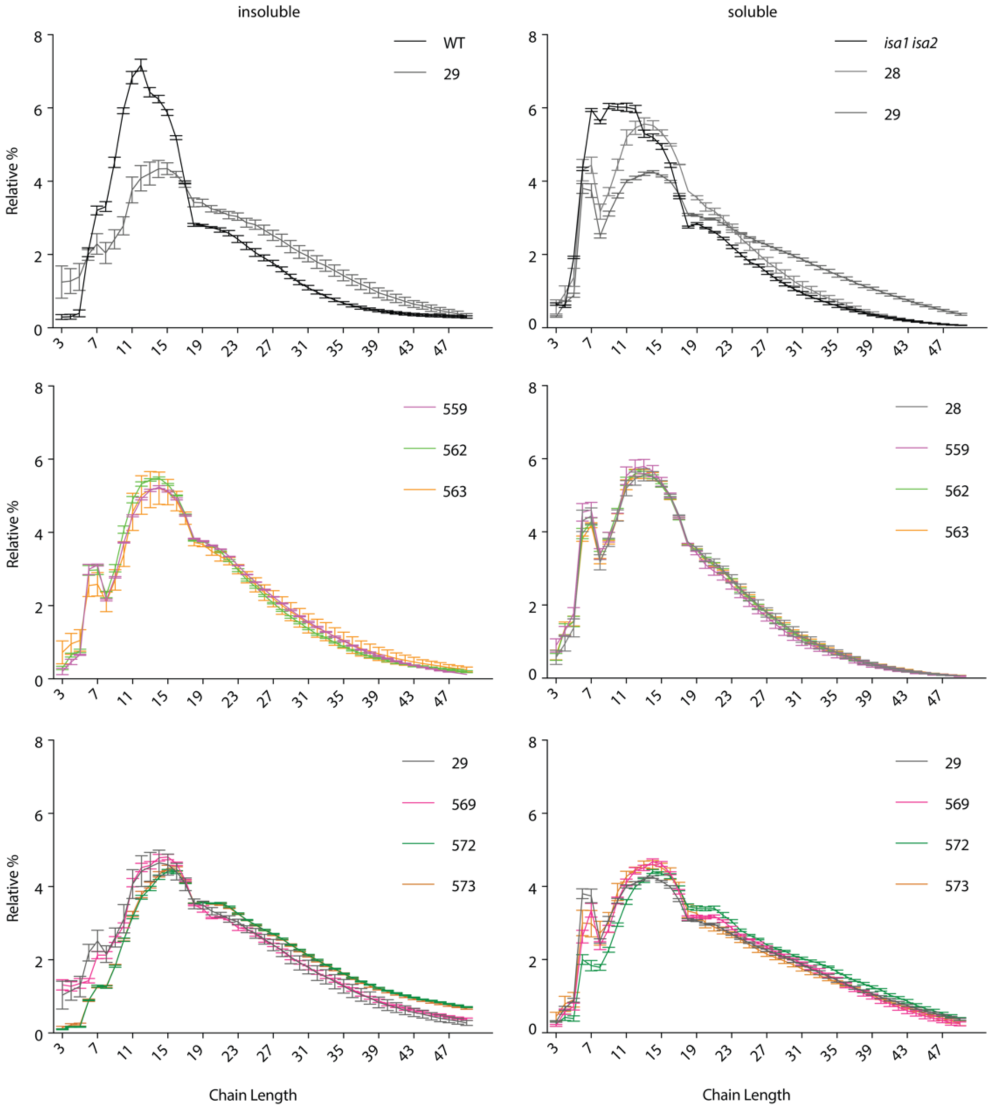
insoluble
soluble
WT
29
isa1 isa2
28
29
559
562
563
28
559
562
563
29
569
572
573
29
569
572
573
Relative %
Relative %
Relative %
Chain Length
Chain Length

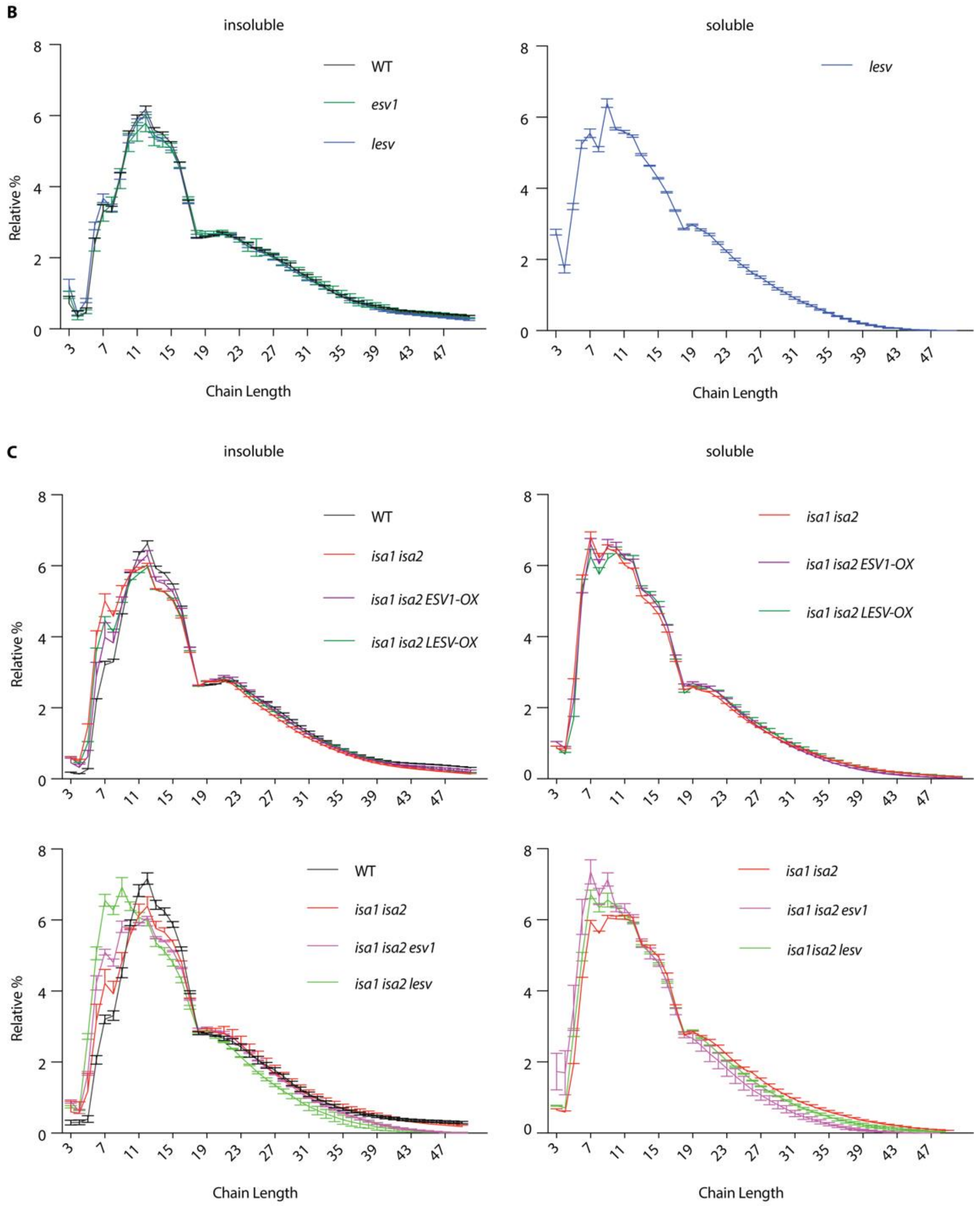

Fig. S5. Fine structure of yeast and plant glucans. Chain length distributions (CLDs) of (**A**) debranched insoluble and soluble glucans purified from the indicated yeast strains, (**B**) *esv1* and *lesv* glucans compared to wild-type Arabidopsis starch (de-starched for 16 h and then harvested after 8 h light), and (**C**) glucans from plant lines harvested after a regular day, compared to wild type (Col-0) Arabidopsis starch and *isa1isa2* phytoglycogen. Values are means ± SE (n=4 independent replicate cultures or biological replicate plants, respectively); relative percentages of values were obtained by dividing peak areas for each chain length by the sum of all analyzed peaks. See Supplementary Text for a discussion of the results.

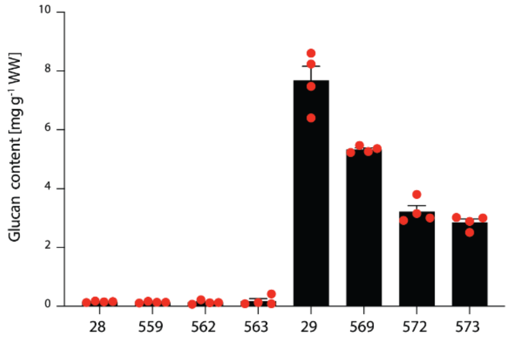

Fig. S6. MOS content of yeast strains grown for 5.75 h in liquid culture under inducing conditions. MOS were measured in the supernatant remaining after methanol precipitation of soluble polyglucans. MOS were enzymatically digested to glucose, which was then quantified using a spectrophotometric assay. Values are means ± SE (*n*=4 independent replicate cultures). See Supplementary Data S2A.

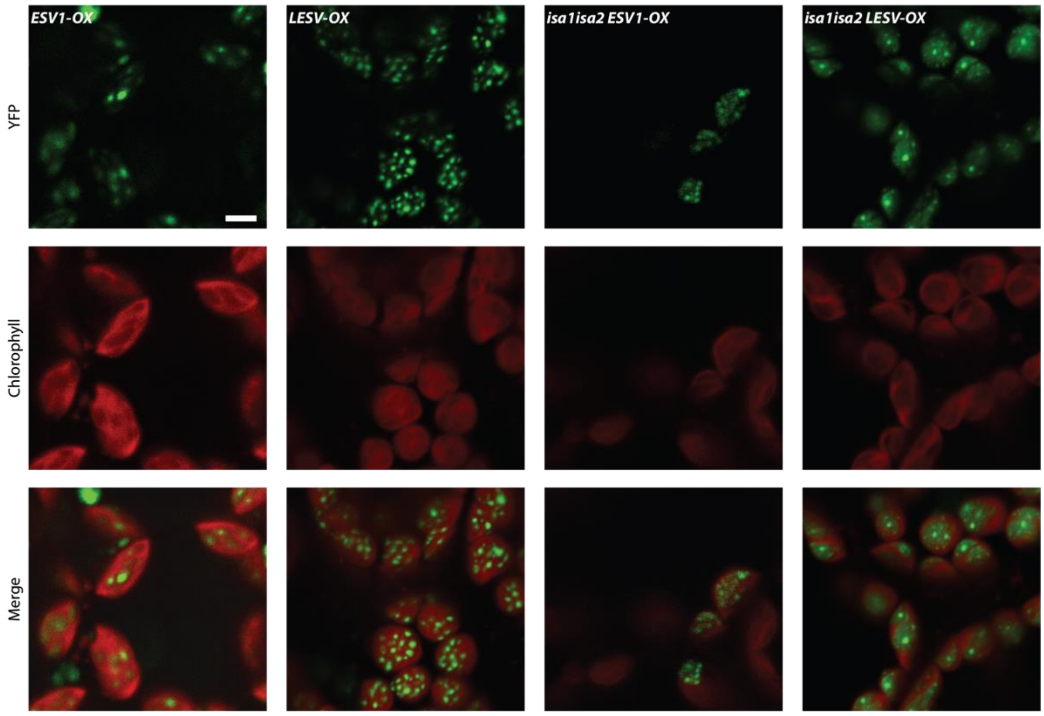

Fig. S7. ESV1 and LESV localization *in planta*. Fluorescent signal in Arabidopsis leaves of lines overexpressing YFP-tagged ESV1 or LESV, imaged at the EOD using confocal microscopy. Scale bar, 5 µm.

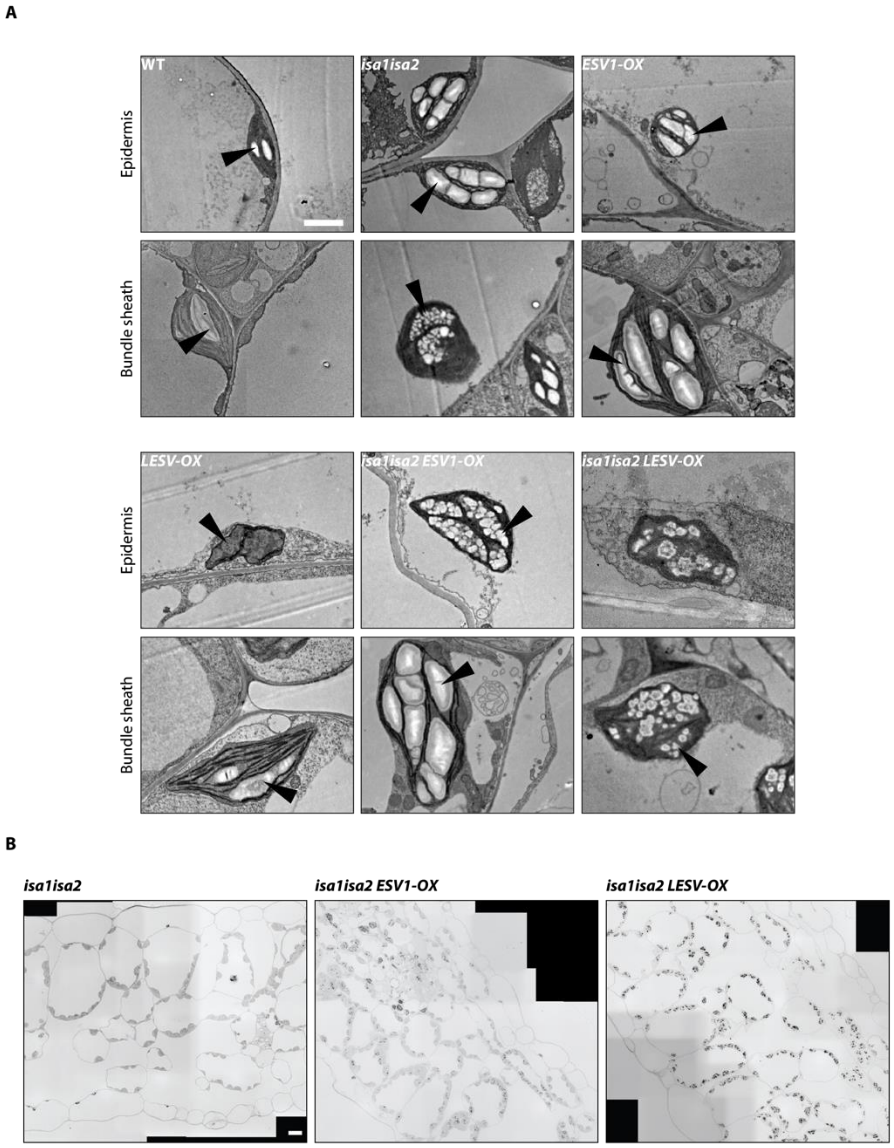

Fig. S8. TEM and overview LM images of ESV1-YFP and LESV-YFP overexpression plants. (**A**) TEM images of epidermal and bundle sheath cells. Black arrowheads indicate starch granules. Scale bar, 2 µm. (**B**) LM overviews, showing starch granules in the chloroplasts of leaf sections prepared from *isa1isa2, isa1isa2 ESV1-OX* and *isa1isa2 LESV-OX*. Individual images were stitched using Fiji to create overviews. Scale bar, 10 µm.

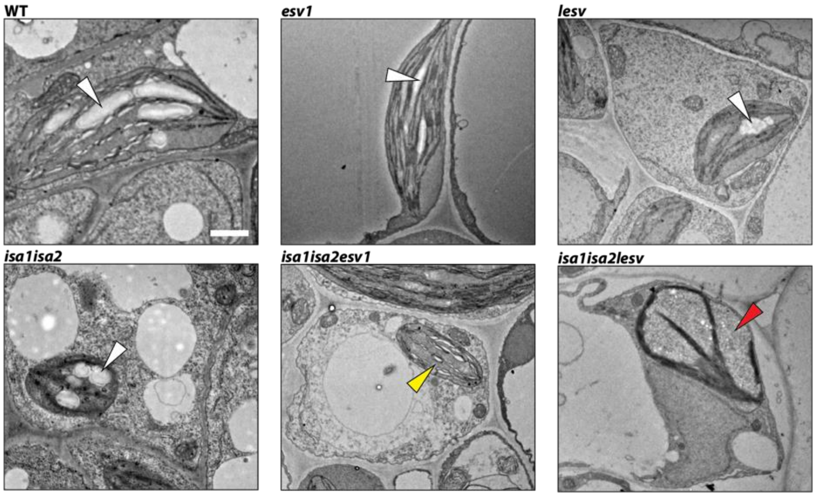

Fig. S9. TEM images of plastids of the bundle sheath. Arrowheads indicate starch granules (white), phytoglycogen (red), and empty plastid sections (yellow). Scale bar, 1 μm.

Table S1. SAXS Structural parameters.

Beamline SWING, SOLEIL	LESV	ESV1
**I(0) [cm^{-1}]**	23.31 +/-0.033	0.031 +/- 5.4e-05
**R_g [Å]**	36.79 +/-0.08	32.03 +/-0.14
**q_{min} [$Å^{-1}$]**	0.014	0.010
**qR_g max**	1.18	1.10
**Coefficient of correlation, R^2**	0.999	0.999
**P(r) analysis**		
**I(0) [cm^{-1}]**	23.82 +/-0.034	0.031 +/-5.8e-05
**R_g [Å]**	39.71 +/-0.102	32.26 +/-0.03
**q range $_{min}$ [$Å^{-1}$]**	0.014 to 0.4	0.010 to 0.25
**Porod volume [$Å^3$]**	121099	83295
**Dmax**	160	115

Table S2. SAXS measurements on purified yeast and plant glucans. Summary of all SAXS measurements for yeast strains and plant genotypes, with the location of the maxima in reciprocal space q_{max} and respective repeat distance d. Replicates in bold are the replicates shown in Figure 9.

**Yeast strain #**	***n***	**Replicate**	**q_{max} [nm^{-1}]**	**d [nm]**
562	*2*	**1**	**0.65**	**9.7**
		2	0.64	9.8
29	*2*	**1**	**0.48**	**13.1**
		2	0.48	13.1
572	*2*	**1**	**0.59**	**10.6**
		2	0.57	11.0
559	*3*	1	0.58	10.8
		2	0.6	10.5
		**3**	**0.59**	**10.6**
573	*1*	**1**	**0.58**	**10.8**
563	*2*	1	0.62	10.1
		**2**	**0.62**	**10.1**
569	*1*	**1**	**0.58**	**10.8**

**Plant genotype**		**Replicate**	**q_{max} [nm^{-1}]**	**d [nm]**
isa1isa2	*3*	1	0.64	9.8
		**2**	**0.64**	**9.8**
		3	0.63	10.0
LESV-OX	*3*	**1**	**0.64**	**9.8**
		2	0.63	10.0
		3	0.63	10
WT	*1*	**1**	**0.61**	**10.3**
esv1	*2*	1	0.59	10.6
		**2**	**0.58**	**10.8**
lesv	*1*	**1**	**0.61**	**10.3**
isa1isa2 ESV1-OX	*2*	**1**	**0.61**	**10.3**
		2	0.61	10.3
ESV1-OX	*1*	**1**	**0.62**	**10.1**
isa1isa2 LESV-OX	*2*	1	0.61	10.3
		**2**	**0.62**	**10.1**

Table S3. Description of yeast strains used in this study.

Strain	Genotype	Source
**28**	*MATa MAL2-8C SUC2 his3Δ gdb1Δ gph1Δ glg1Δ glg2Δ glc3::BE3 XII-2::BE2 gsy1::GlgC-TM X-2::SS1 XI-2::SS2 gsy2::SS3 XII-1::SS4*	(*13*)
**559**	*MATa MAL2-8C SUC2 his3Δ gdb1Δ gph1Δ glg1Δ glg2Δ glc3::BE3 XII-2::BE2 gsy1::GlgC-TM X-2::SS1 XI-2::SS2 gsy2::SS3 XII-1::SS4 XII-5::ESV1 HygR*	This study
**562**	*MATa MAL2-8C SUC2 his3Δ gdb1Δ gph1Δ glg1Δ glg2Δ glc3::BE3 XII-2::BE2 gsy1::GlgC-TM X-2::SS1 XI-2::SS2 gsy2::SS3 XII-1::SS4 XII-5::LESV HygR*	This study
**563**	*MATa MAL2-8C SUC2 his3Δ gdb1Δ gph1Δ glg1Δ glg2Δ glc3::BE3 XII-2::BE2 gsy1::GlgC-TM X-2::SS1 XI-2::SS2 gsy2::SS3 XII-1::SS4 XII-5::ESV1-LESV HygR*	This study
**29**	*MATa MAL2-8C SUC2 his3Δ gdb1Δ gph1Δ glg1Δ glg2Δ glc3::BE3 XII-2::BE2 gsy1::GlgC-TM X-2::SS1 X-4::SS2 gsy2::SS3 XII-1::SS4 XI-2::ISA1-ISA2*	(*13*)
**569**	*MATa MAL2-8C SUC2 his3Δ gdb1Δ gph1Δ glg1Δ glg2Δ glc3::BE3 XII-2::BE2 gsy1::GlgC-TM X-2::SS1 X-4::SS2 gsy2::SS3 XII-1::SS4 XI-2::ISA1-ISA2 XII-5::ESV1 HygR*	This study
**572**	*MATa MAL2-8C SUC2 his3Δ gdb1Δ gph1Δ glg1Δ glg2Δ glc3::BE3 XII-2::BE2 gsy1::GlgC-TM X-2::SS1 X-4::SS2 gsy2::SS3 XII-1::SS4 XI-2::ISA1-ISA2 XII-5::LESV1 HygR*	This study
**573**	*MATa MAL2-8C SUC2 his3Δ gdb1Δ gph1Δ glg1Δ glg2Δ glc3::BE3 XII-2::BE2 gsy1::GlgC-TM X-2::SS1 X-4::SS2 gsy2::SS3 XII-1::SS4 XI-2::ISA1-ISA2 XII-5::ESV1-LESV HygR*	This study

Table S4. Oligonucleotide primer sequences used for ESV1 and LESV cloning for expression in *N. benthamiana*, and mutant Arabidopsis alleles and primer sequences used for their genotyping.

Construct to clone	Primers used to select mutant alleles (shown 5'to 3')	Reference
ESV1-full length CDS<br>(expression of ESV1-YFP)	Fw:<br>GGGGACAAGTTTGTACAAAAAAGCAGGCTTC ACCATGAGCGAAATGGCGG<br>Rv:<br>GGGGACCACTTTGTACAAGAAAGCTGGGTCTT GTGGTTGGTCAGGG	This study
Truncated *ESV1* CDS<br>(expression of YFP-ESV1_Trp-rich-region)	Fw:<br>GGGGACAAGTTTGTACAAAAAAGCAGGCTTC ACCGAAGATGGTAGTAGTTGGTTTAGAG<br>Rv:<br>GGGGACCACTTTGTACAAGAAAGCTGGGTCTC ATTGGATCGAAAGCAACT	This study
LESV-full length CDS<br>(expression of LESV-YFP)	Fw:<br>GGGGACAAGTTTGTACAAAAAAGCAGGCTTC ACCATGGCTTTGCGTTTAGGTGTTTCTATAGGG<br>Rv:<br>GGGGACCACTTTGTACAAGAAAGCTGGGTCGG ACATATCAGAAGGCTTCTTAACGGCT	This study
Truncated *LESV* CDS<br>(expression of YFP-LESV_Trp-rich-region)	Fw:<br>GGGGACAAGTTTGTACAAAAAAGCAGGCTTC ACCGAAGATGGATTGAAGTGGTGGAAGCAAA CG<br>Rv:<br>GGGGACCACTTTGTACAAGAAAGCTGGGTCCT AGGACATATCAGAAGGCT	This study

Gene/AGI code	Mutation type, position	Mutant allele	Primers used to select mutant alleles (shown 5'to 3')	Ecotype	Reference
ESV1<br>*AT1G42430*	T-DNA Insertion in intron 1	*esv1-2*,<br>GABI_031C11	Fw: CTCCAAGGCTTACTGGTCCTC<br>Rv: ATATTGACCATCATACTCATTGC<br>T-DNA: CTACAAATTGCCTTTTCTTATCGAC	Col-0	(*14*)
LESV<br>*AT3G55760*	T-DNA Insertion in intron 1	*lesv-1*,<br>SALK_006713	Fw: CTTTGAGAAGACAGTGGGTGG<br>Rev: ATAAGTGGTGCAGCATTGACC<br>T-DNA: TGGTTCACGTAGTGGGCCATCG	Col-0	(*14*)
LESV-OX	Single copy Insertion in	#4-6	Fw: ACGATCGAATGGTGAGCACTCAATTC<br>Rv: GCTATGTCCAATCCCATCAATCACAGC<br>T-DNA: CGCAATTATACATTTAATACGCG	Col-0	(*14*)
ISA1<br>*AT2G39930*	T-DNA Insertion in exon 13	*isa1-1*,<br>SALK_042704	Fw: GGGACAGCCTATGTGATCTGCC<br>Rv: TGGGAAACCATGAGGGAAACA<br>T-DNA: GCGTGGACCGCTTGCTGCAACT	Col-0	(*21*)
ISA2<br>*AT1G03310*	X-ray Single base pair deletion in exon 1	*isa2-1*	Fw: GGTGACGTATTTACCGATGGA Rev: TGACACTTTGAGCAGCAACC<br>The *isa2-1* amplicon is cut by NlaIV	Col-0	(*21*)

Data S1A. (separate file)

Alignment of ESV1 protein sequences used to create Figure 1A and S1A.

Data S1B. (separate file)

Alignment of LESV protein sequences used to create Figure 1A and S1B.

Data S2. (separate file)

Yeast glucan quantification. Raw data and statistical analysis shown in Figure 2B and S6.

Data S3. (separate file)

Glucan quantification of plants grown under regular 12-h light/12-h dark cycles. Raw data and statistical analysis shown in Figure 4A.

Data S4. (separate file)

Glucan quantification of plants initially grown under regular 12-h light/12-h dark cycles and then subjected to a single prolonged night (16 h dark). Raw data and statistical analysis shown in Figure 4C.

Data S5. (separate file)

Maltose quantification of plants initially grown under different diel cycle regimes. Raw data and statistical analysis shown in Figure 4E and F.

Data S6. (separate file)

Glucan quantification of *ESV1-OX* and *LESV-OX* plants. Raw data and statistical analysis shown in Figure 5C.

Data S7. (separate file)

Glucan quantification of triple mutant plants. Raw data and statistical analysis shown in Figure 7B.

Data S8. (separate file)

Plasmids and primers used for heterologous gene expression in yeast.
